# Detection and Phylogenetic Analysis of an Exotic Strain of Porcine Epidemic Diarrhea Virus and Its Effect on an Affected Herd Immunized Against the Endemic Strain in Thailand

**DOI:** 10.3390/ani15020225

**Published:** 2025-01-15

**Authors:** Christopher James Stott, Patumporn Jermsutjarit, Pornchai Pornpanom, Hongyao Lin, Angkana Tantituvanont, Dachrit Nilubol

**Affiliations:** 1 Akkhararatchakumari Veterinary College, Walailak University, Nakhon Si Thammarat 80160, Thailand; christopher.st@wu.ac.th (C.J.S.); pornchai.po@wu.ac.th (P.P.); 2 One Health Research Centre, Walailak University, Nakhon Si Thammarat 80160, Thailand; 3 Swine Viral Evolution and Vaccine Development Research Unit, Department of Veterinary Microbiology, Faculty of Veterinary Science, Chulalongkorn University, Bangkok 10330, Thailand; 4 Center of Excellence in Informatics Innovation, Walailak University, Nakhon Si Thammarat 80160, Thailand; 5 MSD Animal Health Innovation Pte Ltd., Singapore 718847, Singapore; 6 Department of Pharmaceutics and Industrial Pharmacy, Faculty of Pharmaceutical Sciences, Chulalongkorn University, Bangkok 10330, Thailand

**Keywords:** PEDV, coronavirus, swine, phylogenetic, evolution, MDA, Thailand

## Abstract

Porcine epidemic diarrhea (PED) is caused by the PED virus, with **genogroup 2** (**G2**), comprising **G2a** and **G2b**, being the primary contributors to outbreaks worldwide. In Thailand, **G2a** was the only virulent strain identified until the emergence of the first **G2b** strain in 2014, followed by additional **G2b** strains in 2015–2016, particularly those closely related to virulent strains from China. This led to increased awareness of more frequent and complex outbreaks. This study examined farms affected by **G2a** and **G2b** strains, both employing planned exposure of sows to intestines from **G2a**-affected piglets, a traditional practice in Thailand. The analysis focused on the milk and colostrum profiles of sows, including viral neutralization and ELISA methods, as well as histopathological assessments of piglet intestinal villi. Additionally, a novel **G2b** strain was sequenced for phylogenetic and protein structure analyses. The results revealed no significant differences in colostrum and milk profiles between the farms. However, piglets infected with **G2b** showed significantly more severe intestinal damage. A unique insertion in the protein structure of the novel **G2b** strain led to the identification of a new epitope and potential changes in viral properties. This study highlights the importance of monitoring emerging strains and provides valuable insights for enhancing preventive strategies and advancing phylogenetic research.

## Simple Summary

Porcine epidemic diarrhea is a contagious disease, causing watery diarrhea globally and resulting in significant piglet production losses. In regions where external PEDV strains have not been introduced, planned exposure has proven to be an effective method of disease control. However, this approach has been less effective in regions affected by multiple PEDV strains. In Thailand, **genogroup 2a** remained the dominant strain until 2014, when **genogroup 2b** emerged in certain regions. Since its introduction, outbreaks have become more frequent despite the continued use of planned exposure. This study aims to investigate the factors contributing to the ongoing outbreaks after the large-scale outbreak in Thailand during 2014–2015.

## 1. Introduction

Porcine epidemic diarrhea virus (PEDV) is the causative agent of porcine epidemic diarrhea (PED) in pigs. While the infection can occur at any age, mortality is significantly higher in piglets. Clinical signs include vomiting, watery diarrhea, and severe dehydration, and they often result in death [1,2,3]. The primary route of transmission is the fecal–oral route, with replication occurring in intestinal epithelial cells. Although PEDV can be detected in sow milk and alveolar macrophages, it is possible that the virus also spreads through vertical and respiratory transmission routes [4]. The severity of PEDV infection can be assessed through histopathological examination of villous atrophy. PEDV initially damages the villous epithelium, causing shortening of the villi. As the infection progresses, the villi become increasingly atrophied, reducing the absorptive surface area and exacerbating clinical signs such as diarrhea. Histopathological analysis of these atrophic changes offers a clear evaluation of infection severity and provides valuable insight into the pathogen’s overall impact [5].

PEDV is an enveloped, positive-sense, single-stranded RNA virus in the subgenus *Pedacovirus*, genus *Alphacoronavirus*, family *Coronaviridae*, order *Nidovirales* [6]. Its genome is approximately 28,000 bases, comprising seven open reading frames (ORFs), including ORF1a/b, spike (S), ORF3, envelope, membrane, and nucleocapsid protein [7]. PEDV is globally classified into two genogroups, **G1** and **G2**; **G2** can also classified based on spike gene sequences, including **G2a**, **G2b**, **G2c/S-INDEL**, and recombinant genotypes. G2a is endemic to Asia and includes strains identified in South Korea, China, and Thailand more than a decade ago [8,9]. **G2b** is associated with widespread outbreaks worldwide, including China-like strains primarily found in China, Vietnam, and Thailand, as well as US-like or global strains that have spread to most PEDV-affected countries. The S protein helps the virus attach to host–cell receptors and is made up of two subunits: S1 (amino acids 1–725) and S2 (amino acids 726–1383) [8,10]. This protein is widely studied to understand the genetic relationships between PEDV strains.

According to previous reports, PEDV’s S protein has four primary antigenic epitopes: the CO equivalent (COE) domain (amino acids 499–638), SS2 (amino acids 748–755), SS6 (amino acids 764–771), and 2C10 (amino acids 1368–1374) [10,11,12]. A novel neutralizing epitope, named A3, was recently identified in Thailand [13]. Recent studies on PEDV in China [10] highlighted extensive variation in the spike gene epitopes, including the signal peptide (SP) and S1-N-terminal domain (S1-NTD), which exhibit high variability in circulating strains. Additionally, based on comparisons with HCoV NL63, the S1 subunit can be divided into distinct regions: SP (amino acids 1–18), subdomain 0 (amino acids 19–219), subdomain A (amino acids 220–509), subdomain B (amino acids 510–639), and subdomain CD (amino acids 640–728) [14].

In Thailand, PED was first recognized in 1995, and the pathogen was confirmed in 2007, with the responsible variant classified as **genogroup G2a** [15]. Since then, **G2a** PEDV has continued to cause outbreaks [9]. In 2014, **genogroup G1** was first discovered in Thailand in **G1** intramuscular-vaccinated herds in the Eastern region [16]. The outbreak situation in Thailand has become more complex, with reports of novel and exotic strains, including **TH3-G2a**, **TH4-G2b**, **TH5-G1a**, and **TH6-G2b**, leading to recurrent outbreaks across multiple farms [9]. Planned exposure of sows using feedback material has been effective in controlling the pathogen in areas without the introduction of external strains. However, the emergence of exotic strains and co-infections with multiple genogroups in several regions has made control efforts more challenging.

In 2015, a PEDV strain in Thailand with an insertion in a similar position on the spike gene, as previously reported [9], was discovered. This strain (accession number MZ090589), classified as either **G2a** or **G2b**, was identified as an exotic strain in Thailand and was possibly a recombinant strain between a Thai endemic strain and a China-like **G2b** strain. This finding raised concerns that the China-like **G2b** strain might have been introduced as a field strain in Thailand, following its earlier link to an outbreak in Vietnam (CBR1 and CBR2) in 2014 [16]. A year later, another China-like **G2b** strain (accession number LC496368) was reported by a different research group in Thailand. Unlike the CBR strain from 2014, this strain was directly related to the **G2b** strain spreading in China. Both strains were closely associated with the **G2b-TH4** genogroup (China-like strains).

Between 2016 and 2017, sporadic PEDV outbreaks were reported. After 2017, however, evidence of virus outbreaks increased, although the incidence remained lower than the large-scale outbreaks of 2014–2015, which were dominated by the **G2a** strain. This trend suggests the possible introduction of exotic strains distinct from the endemic strain or mutations occurring within Thailand, as most outbreak viruses used for planned exposure to raise maternally derived antibodies (MDAs) in sows belong to **G2a**.

In 2019, despite a decrease in PEDV incidence, possibly due to enhanced biosecurity measures against highly contagious viruses like African Swine Fever in Southeast Asia, an outbreak occurred in a stable herd in central Thailand. This farm employed planned exposure using feedback material from **G2a**-affected swine intestines to control the disease. However, the situation remained unstable, and detecting the pathogen through RT-PCR proved challenging. Partial spike gene sequencing suggested that the virus belonged to the **G2b** strain. Under the supervision of one of our colleagues, we initiated a study to sequence the full genome of this strain by designing specific primers. This approach aims to provide insights into the **G2b** strain in Thailand and its interaction with **G2a** (endemic; **TH1**) and **G2b** (exotic; **TH4**) strains.

The objectives of this study were to investigate the underlying pathogens related to persistent or recurrent PEDV outbreaks in experienced herds, combining laboratory diagnostic data, including antibody responses, viral neutralization, and immunoglobulins A and G in colostrum and milk, using planned exposure against the same Thailand endemic strain (**G2a**). We observed two different farms in different provinces, one affected by **G2a** and the other by **G2b**, to determine the effectiveness of planned exposure against the virus. This was assessed by examining histopathological lesions in piglet villi and comparing the protein structure and epitope predictions with previously discovered strains in Thailand. The study results could provide valuable insights into the introduction of exotic strains, aid in developing preventive strategies against PEDV, and contribute to phylogenetic studies.

## 2. Materials and Methods

### 2.1. Ethics Statement

All animal procedures were conducted in accordance with the recommendations in the Guide for the Care and Use of Laboratory Animals of the National Research Council of Thailand and approved by the Faculty of Veterinary Science, Mahidol University-Institute Animal Care and Use Committee (FVS-MU-IACUC; animal use license number U1-01281-2558). The handling of samples and the performance of virus sequencing in the SVEVR unit were conducted under the Institutional Biosafety Committee (IBC) of the Faculty of Veterinary Science, Chulalongkorn University (#2231028).

### 2.2. Specimen Isolation and Sequencing

The samples submitted for detection included colostrum and milk from sows (*n* = 30) to assess the immunization levels against **TH1-G2a**. These samples were collected from Farm A (**G2b**-affected group; *n* = 10), Farm B (**G2a**-affected group; *n* = 10), and Farm C (control or negative status; *n* = 10). Additionally, intestinal tissues were collected from piglets on the farms immediately after death. Intestines from piglets that died 2–3 days post-farrowing (DPF) in each pen of each farm (*n* = 30) were associated with watery diarrhea and confirmed to be infected with PEDV using in-house reverse transcription quantitative polymerase chain reaction (RT-qPCR) with SYBR Green (Thermo Fisher Scientific, Waltham, MA, USA). Histopathological examination was conducted to evaluate the severity of infection. The primers used for PEDV detection were as follows: Forward: 5′-ACA GCT TCC CAG CGT AGT-3′ and Reverse: 5′-GAT CTG GAC CTG TTG TTG CC-3′. All piglets tested negative for transmissible gastroenteritis virus (TGEV), porcine deltacoronavirus (PDCoV), and Rotavirus A.

Samples were collected from sows at parity 2–4 from herds experiencing **G2a**-PEDV (*n* = 10; Farm A) and **G2b**-PEDV (*n* = 10; Farm B). According to farm management protocols, gilts were acclimatized for 7 days before their integration into the herd and were fasted prior to the oral administration of feedback materials. Two weeks before farrowing, the sows were administered 10 g of homogenized intestines from PEDV-infected piglets containing PEDV at a dosage of 10^3.8^ TCID_50_/mL of the endemic strain. After farrowing, colostrum samples were collected within 3 h, and milk samples were collected 3 days post-farrowing (DPF). Colostrum and milk samples were assayed for PEDV-specific antibodies using in-house ELISA for IgG and IgA, as well as a viral neutralization (VN) assay.

The virus from Farm A was confirmed as the same strain as **TH1** (with the insertion), as described in a previous study reported by our laboratory, while the pathogen from Farm B was classified as **TH4** (China-like) [9,15]. Intestinal samples from Farm B were prepared and stored for polymerase chain reaction (PCR) procedures for further study as reported previously by our laboratory [9,15]. Full-length genome sequencing was performed using primers described in prior studies [15,17,18]. If any sequences were missing or substituted, the primers were redesigned.

### 2.3. Phylogenetic Analysis

All available full-length PEDV genomes with at least 95% coverage of the reference genome (NC_003436) (*n =* 849; obtained on 30 June 2024) were retrieved from GENBANK^®^ [19]. The sequences were then ordered by year, month, and date (yyyy-mm-dd) of sample collection. Missing data were filled with “NA” (not available) and subjected to CD-HIT at 99% identity, grouped separately by country to obtain the earliest representative sequences for phylogenetic study. Missing months or dates were replaced with the middle of the year or month, respectively. The full-length genomes obtained in this study were aligned with the references using MAFFT (v7.520) [20] to create a comprehensive dataset of full-length genomes. These sequences were further aligned with previously representative sequences from Thailand in GENBANK^®^ to locate the **G2a** clade of PEDV in Thailand, creating a spike gene dataset. Both datasets were used to construct a phylogenetic tree using IQ-TREE (version 1.6.12) and FigTree (version 1.4.4) [21,22], applying a best-fit model (GTR+G4+I+F) and performing 1000 bootstrap replicates. The resulting phylogenetic tree was analyzed using TempEst (v1.5.3) [23] to exclude outliers by year, and the tree was re-evaluated after these adjustments. Additionally, an identity matrix was analyzed to identify closely related sequences based on nucleotide identity and amino acid similarity using BioEdit (v5.0.9) [24].

### 2.4. Serological Analysis of Milk and Colostrum Samples

Colostrum and milk samples were assayed for PEDV-specific antibodies using viral neutralization (VN) and ELISA for IgG and IgA, assayed as previously described protocols [25]. Briefly, colostrum and milk samples were centrifuged at 8000× *g* for 20 min at 4 °C. The lipid layer was removed, and the middle layer was collected and stored at −20 °C for antibody detection analysis. For VN, heat-inactivated colostrum, milk, and serum samples were two-fold serially diluted from 1:4 to 1:512. Each dilution was mixed with an equal volume of PEDV diluted to contain 100 TCID_50_/0.1 mL. The sample–virus mixtures were incubated at 37 °C in a 5% CO_2_ incubator for 1 h. The mixtures were then added in duplicate to the wells of a 96-well microtiter plate containing a 48 h old confluent Vero cell monolayer and incubated at 37 °C in a 5% CO_2_ incubator for an additional 3 days.

After incubation, the cell monolayers were fixed with a cold acetone–methanol solution, and the presence of the virus was detected using indirect fluorescent microscopy following the fluorescent focus neutralization (FFN) protocol. A fluorescein isothiocyanate (FITC)-conjugated anti-PEDV N protein monoclonal antibody (SD6-29, Medgene Labs, Brookings, SD, USA) diluted in PBS (1:100) was added to each well and incubated at 37 °C in a 5% CO_2_ incubator for 30 min. The titers were expressed as the reciprocal of the highest serum dilution that resulted in a 90% reduction in the number of fluorescent foci compared to control wells under a fluorescent microscope. All assays were performed in duplicate, and results were displayed as geometric mean titers.

For ELISA, microtiter plates were coated with recombinant spike protein and incubated overnight at 4 °C. After blocking with skim milk, plates were incubated with diluted colostrum and milk. Following washing, anti-pig IgG-HRP or IgA-HRP was added, and after further washing, TMB substrate was used for detection. The reaction was stopped with sulfuric acid, and optical density at 450 nm was measured. Controls included negative and positive samples from PED-free and PED outbreak herds. Antibody responses were calculated as sample-to-positive (S/P) ratios.

### 2.5. Histopathological Examination

The duodenum, jejunum, and ileum were retrieved from the intestinal samples. Histopathological examination was performed according to standard procedures. The integrity of the epithelial cell lining was measured through villous height at the duodenum, jejunum, and ileum using a computerized image system (Olympus cellSens^TM^ digital image software v1.16). The means of villous height and villous-height-to-crypt-depth ratio (VCR) from each intestinal segment, used to determine the differences between groups, were generated from 5 areas in each histopathological section and the analysis of each segment of the small intestine was performed in quadruplicate.

### 2.6. Protein Analysis

The protein structures and epitope predictions were performed using the following sequences: **G2a** from Thailand (KC764953; 2008), **G2a** from this study (KX981897; 2014), previously identified **G2b** strains (KR610993; 2014 and LC496368; 2016), **G2b** recombinant (MZ090589; 2015), and **G2b** from this study. The SWISS-MODEL application [26] was used for protein structure simulation, with hydrophobicity visualized using Wimley–White’s scale [27] and interpretation performed with iCn3D [28]. At this stage, **G2** sequences containing insertions in the spike gene from the representative dataset were displayed to compare the insertion sites. Epitope predictions, including both linear and discontinuous epitopes, were conducted using ElliPro (http://tools.iedb.org/ellipro/, accessed on 20 December 2024). [29] based on the constructed protein structures described earlier. Manipulation of the VCF and DTI1 protein structures, as well as exporting of the models, was performed using UCSF Chimera (version 1.18) [30] for displaying movable 3D structures.

### 2.7. Data Analysis

All parametric data, including parities, viral neutralization titers, immunoglobulin A and G levels, villous length, and the villous-height-to-crypt-depth ratio, were analyzed using analysis of variance (ANOVA) across the observed farms in R (version 4.4.1) [31], with a significance level of *p* < 0.05.

## 3. Results

### 3.1. Specimen Characteristics and Sequencing Outcomes

All samples from sows were collected and subjected to the tests described above. However, two intestinal samples from Farm A and three from Farm B which were submitted for histopathological analysis were excluded from the statistical analysis due to tissue degradation.

Four full-length genomes were retrieved from specimens at Farm B, including DTI1, DTI2, DTI3, and DTI4 (DTI1–4), using previously described primers along with some redesigned primers, as detailed in Table S1. All genomes were deposited in GENBANK^®^ under accession numbers MW805354 to MW805357 for further study.

### 3.2. Phylogenetic Interpretation of PEDV

**Figure 1:**
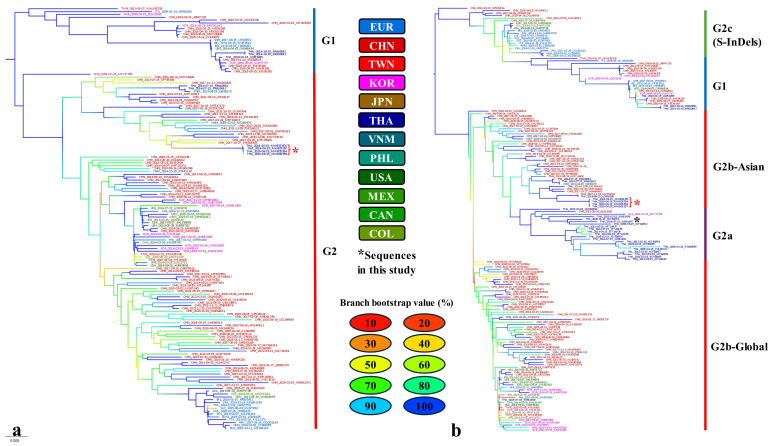
(**a**) Phylogenetic tree of the full-length genomes and (**b**) phylogenetic tree of the spike genes from all full-length genomes and Thailand PEDV sequences available in GenBank^®^, constructed using IQ-TREE (v2.3.6) with 1000 bootstraps. Three-letter abbreviations represent countries, as indicated by the tip label colors. The blue and red lines on the right side of left panel demonstrate genogroups 1 (**G1**) and 2 (**G2**). Furthermore, on the right panel, **G2a**, **G2b** and **G2c** are represented in purple, red and green, respectively. The branch colors represent approximate confidence levels, determined by bootstrap values, ranging from red to blue based on hex color codes. Red brackets with asterisks denote the sequences acquired in this study (DTI1–4; MW805354-MW805357). Black asterisks denote the representative strain of the strain discovered in another farm in this study (KX981897).

A total of 148 sequences were selected as representatives for the full-length genome dataset, while 21 additional sequences were included in the spike gene dataset (Table S2). Phylogenetic trees were constructed and are displayed in Figure 1, with bootstrap values used for branch labeling. According to IQ-TREE analysis, the spike gene data suggest that **genogroup 2** can be further divided into four clusters: **G2a, G2b-Asian**, **G2b-Global**, and **G2c** (or **S-InDels**).

The phylogenetic tree indicates that DTI1–4 is located within the monophyletic **G2** group, which includes several China PEDV strains, in both the complete genome and spike gene trees (Figure 1). However, the complete genome tree shows a lower bootstrap value for the clade containing CHN/MK644603 compared to the spike gene tree. The spike gene tree shows that DTI1–4 are strongly related to CHN/MZ364310, CHN/MH726406, CHN/MK644603, CHN/MZ364311, and THA/LC496368.

Based on the complete genome identity matrix, the sequences in this study (DTI1–4) share 99.9% identity with each other. They are closely related to CHN/MH726403 with 98.7% identity and have 97.6% identity with the previously identified **TH4-G2b** from Thailand (THA/KR610993).

The spike gene identity matrix indicates that DTI1–4 share 99.9–100% identity and 100% similarity with each other. They are closest to THA/LC496368 and CHN/MZ364311, with 97.7% identity and 98% similarity. Compared to Thailand PEDV representative sequences, DTI1–4 share 93.5% to 95.5% identity and 92.7% to 95.5% similarity with **TH1-G2a**, **TH2-G2a**, and **TH3-G2a**, while sharing 96.4% to 96.6% identity and 95.9% to 96.3% similarity with the previously identified **TH4-G2b** from Thailand.

### 3.3. Viral Neutralization and ELISA

A comparison between the three farms demonstrated that most parameters were not significantly different. Specifically, there was no difference between Farms A and B, while some differences were observed between Farm C and Farm A (IgA at 1 DPF) and between Farm C and Farms A and B (VN and IgG at 3 DPF). The descriptive statistics are as follows: the parity of sows in Farms A, B, and C was 3.1 (±0.738), 3.2 (±0.422), and 3.05 (±0.600), respectively. The VN titers at 0 days post-farrowing (DPF) were 3.4 (±0.966) for Farm A, 3.5 (±0.972) for Farm B, and 3.45 (±0.950) for Farm C. At 3 DPF, the VN titers were 2.7 (±1.059) for Farm A, 2.9 (±0.876) for Farm B, and 3.0 (±0.900) for Farm C.

**Figure 2:**
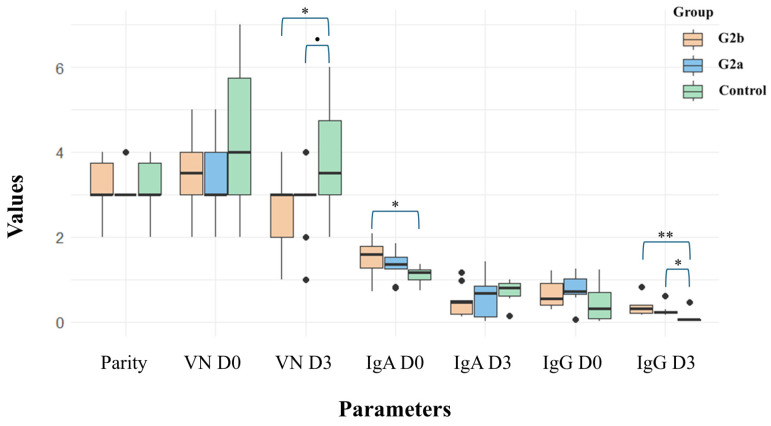
The parity, geometric mean titer (GMT) of the viral neutralization activity of colostrum and milk of sows at 0 (VN D0) and 3 (VN D3) days post-farrowing (DPF), the S/P ratio of immunoglobulin A (IgA) at 0 (IgA D0) and 3 (IgA D3) DPF, and immunoglobulin G (IgG) at 0 (IgG D0) and 3 (IgG D3) DPF of the colostrum and milk of sows. Orange, blue, and green colors represent samples from **G2b**-affected (**G2b**), **G2a**-affected (**G2a**), and negative status (control) sows, respectively. The range of *p*-values is indicated by asterisks (*** for 0–0.001, ** for 0.001–0.01, and * for 0.01–0.05) and small dots (0.05–0.1), above the brackets, which denote significant differences between groups. Black dots represent outlier data in the boxplot.

The ELISA S/P ratio of IgA in colostrum and milk at 0 and 3 DPF was 1.506 (±0.446) and 0.471 (±0.355), respectively, for Farm A; 1.337 (±0.335) and 0.599 (±0.501), respectively, for Farm B; and 1.450 (±0.400) and 0.500 (±0.400), respectively, for Farm C. The ELISA S/P ratio of IgG in colostrum and milk at 0 and 3 DPF was 0.658 (±0.332) and 0.341 (±0.191), respectively, for Farm A; 0.775 (±0.345) and 0.258 (±0.125), respectively, for Farm B; and 0.700 (±0.300) and 0.300 (±0.200), respectively, for Farm C (Figure 2 and Table S3).

### 3.4. Histopathological Findings

**Figure 3:**
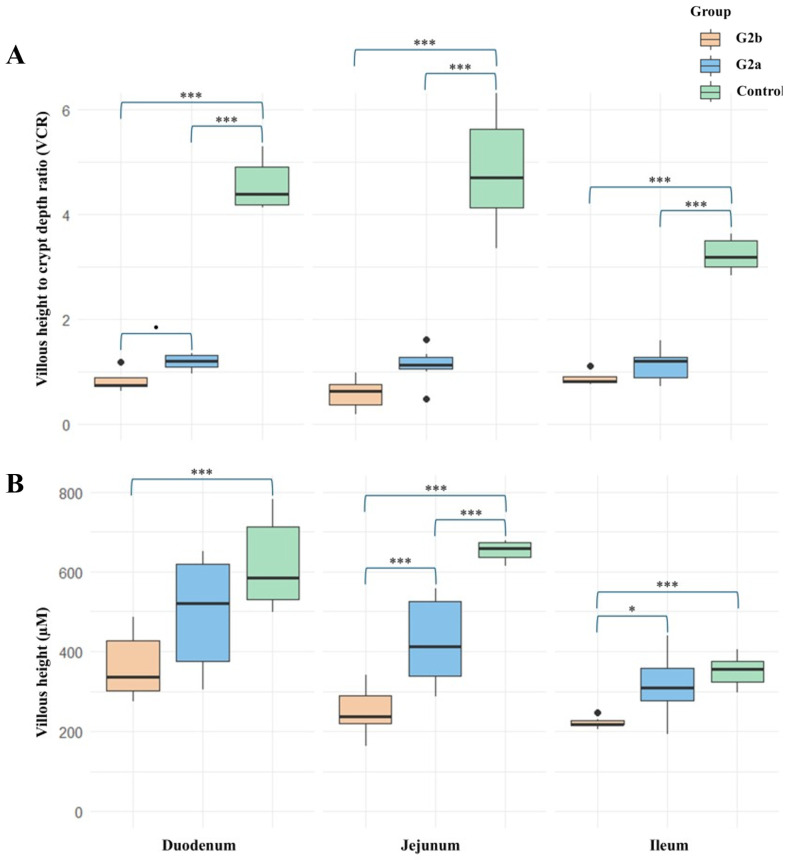
(**A**) The villous-height-to-crypt-depth ratio (VCR) and (**B**) villous length of piglets’ duodenum, jejunum, and ileum were determined using histopathology of the intestinal segments. The range of *p*-values is indicated by asterisks (*** for 0–0.001, ** for 0.001–0.01, and * for 0.01–0.05) and small dots (0.05–0.1), which denote significant differences between the affected piglets. Black dots represent outlier data in the boxplot.

The VCR values of the small intestines of piglets are shown in Figure 3. Briefly, the VCR values for Farms A and B were highly significantly lower (*p* < 0.001) than those of Farm C, while the duodenal VCR of Farm A was marginally significantly lower than that of Farm B (*p* < 0.1). The villous height of the duodenum in Farm A was highly significantly lower (*p* < 0.001) than that of Farm C, while no significant difference was observed between Farms B and C. The villous height of the jejunum in Farm A was highly significantly lower than those of Farms B and C. However, the villous height in Farm B was also highly significantly lower than that of Farm C. For the ileum, the villous height in Farm A was significantly lower than that of Farm B (*p* < 0.05) and highly significantly lower than that of Farm C (*p* < 0.001) (Figure 3 and Table S4).

### 3.5. Protein Structure and Epitope Prediction

**Figure 4:**
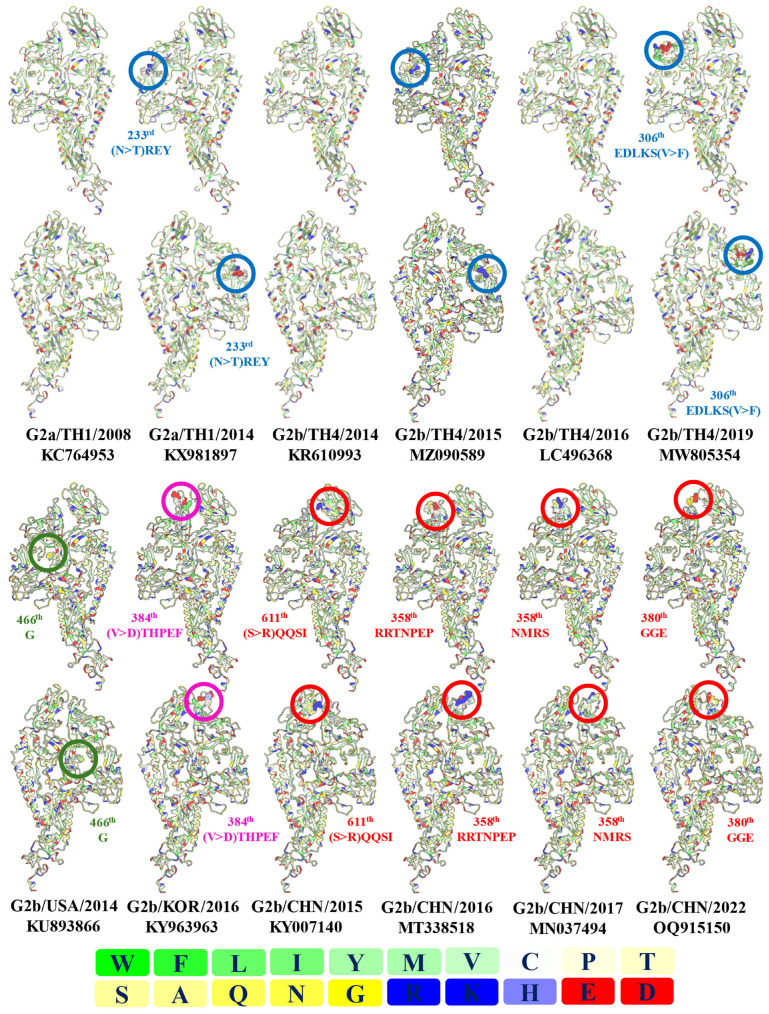
The protein structures of NPPED (G2a/TH1/2008; KC764953), VCF with insertion (G2a/TH1/2014; KX981897), CBR1 (G2b/TH4/2014; KR610993), C9822-GEN (G2b/TH4/2015), BP-2016 (G2b/TH4/2016; LC496368), and DTI1 (G2b/TH/2019; MW805354) from Thailand are compared to the predicted spike structures from other regions with insertions, including PC22A-P50 (G2b/USA/2014; KU893866), KNU-1601 (G2b/KOR/2016; KY963963), LNsy (G2b/CHN/2015; KY007140), HNAY2016 (G2b/CHN/2016; MT338518), WHLL (G2b/CHN/2017; MN037494), and ZJ2022 (G2b/CHN/2022; OQ915150). These structures were predicted using SWISS-MODEL and visualized with the Wimley–White Hydrophobicity Scale in iCn3D (v3.40.0). The color chart on the right-hand side indicates the residues based on the hydrophobicity scale. The blue, green, pink, and red circles highlight the amino acid insertion sites in the representative structure, corresponding to the insertions in Thailand, the US, South Korea, and China, respectively.

According to structure prediction by SWISS-MODEL, the template SMTL ID: 7w6m (Pintung 52) [32] was suggested. The protein structures of NPPED2008 (KC764953), VCF (KX981897), C9822 (MZ090589), CBR1 (KR610993), BP-2016 (LC496368), and DTI1 were constructed. Among these, major differences were observed in VCF and DTI1 due to insertions. Specifically, in VCF, the amino acid changed from N to T at position 233, followed by the insertion of REY at this site. In DTI1, there was an insertion of EDLKS at position 306, along with an amino acid change from V to F (Figure 4).

Some global strains with insertions were also modeled, including those from the US (KU893866, 466th G), South Korea (KY963963, 384th (V > D)THPEF), and China (KY007140, 611th (S > R)QQSI; MT338518, 358th RRTNPEP; MN037494, 358th NMRS; and OQ915150, 380th GGE).

For the linear epitope prediction, DTI1 shows the absence of epitopes between positions 31–38 and 42–46 compared to other **G2b** strains like BP-2016 and CBR1. This finding aligns with VCF, which also has an insertion. Specifically, the amino acid at position 43 is S in VCF, whereas it is A in the other strains. Most of the remaining epitopes are similar, although some changes in properties were noted. Notably, there is an increase in the epitope at positions 305–307 (GEDLKSFC) due to an insertion at this site in DTI1 (Table S5).

The discontinuous epitope (DE) prediction demonstrates the following epitopes:

- DE1, which covers approximately amino acids 773–1258 (167–199 residues);
- DE2, which covers approximately amino acids 497–702 (192–195 residues);
- DE3, which covers approximately amino acids 1061–1093 (6–23 residues);
- DE4, which covers approximately amino acids 287–442 (76–77 residues);
- DE5, which covers approximately amino acids 31–314 (114–117 residues);
- DE6, which covers approximately amino acids 1061–1093 (6–23 residues);
- DE7, which covers approximately amino acids 879–897 (12 residues); and
- A novel DE8, which covers approximately amino acids 301–310 (8 residues).

All predicted strains except CBR contain DE3, while all strains except C9822 contain DE6, although VCF has a shorter DE6. Interestingly, DE3 in VCF and C9822 have a higher number of residues (49 and 54, respectively) compared to other strains (31). This increase correlates with a reduction in the number of residues in DE6 in VCF (6 residues compared to 20–23 residues) and the absence of DE6 in C9822.

Additionally, DTI1 contains a novel epitope, DE8, which consists of amino acids 301–302 (QT) and 305–310 (GEDLKS), totaling eight residues. This site corresponds to the same site identified in the linear epitope prediction (Tables S5 and S6) and also contains the unique insertion characteristic of this strain.

## 4. Discussion

In this study, we redesigned the primers (Table S1) to detect a novel strain in Thailand and conducted an analysis using representative sequences acquired from all available full-length sequences as described previously. To reduce phylogenetic error, we excluded two outlier sequences based on TempEst analysis: Yorkshire/2000 (accession number KU836638) and Belgorod/2008 (accession number MF577027). Due to the lack of available full-length **G2a** sequences in GENBANK^®^, we constructed an additional phylogenetic tree by including the representative dataset of Thailand PEDV available in GENBANK^®^. Although **G2b** diverges from **G2a**, results from IQ-TREE suggest that **G2a** and **G2b-Asian** may share a common ancestor. Many authors have classified China strains into different clusters within G2 [33,34], but since China strains outside **G2b-Asian** might be influenced by **G2b-Global** (such as US-like strains), we classified them together in **G2b-Global**. For S-InDels, we classified them as **G2c** based on full-length genome data, although some authors have classified them as **G1b** [8]. Although DTI1 was identified as China-like **G2b**, the closest strain was found in Thailand in 2016, followed by China in 2017. This suggests that China PEDV might also be influenced by PEDV strains in Southeast Asia, including Thailand. Further investigations using more effective phylogenetic methods, such as Bayesian analysis on a larger scale, should be conducted.

The results from the piglet intestinal samples strongly indicate that piglets affected by the DTI strain experience more severe disease compared to those affected by the VCF strain. This observation is consistent across piglets with maternally derived antibodies (MDA) from sows immunized using planned exposure with **G2a**-affected feedback materials. Both farms had similar levels of immunization, with no significant differences noted, despite the considerable differences between the DTI and VCF strains, especially at the insertion site. Our experience, along with previous reports, suggests that the level of immunization achieved through planned exposure can vary and may contribute to the rapid evolution of the virus [35,36], and the strains affecting herds may also differ. For instance, although **TH1-G2a** falls within the same cluster, variations such as the presence or absence of insertions warrant further investigation. This study was limited to examining samples submitted from farms to our laboratory, which restricts our ability to assess all influencing factors comprehensively. Nonetheless, we can confirm that PEDV strains from different clusters within the same genogroup can cause more severe damage to piglet populations, potentially worsening the PEDV situation in affected herds.

Although the Thailand endemic strain used in planned exposure is classified within the **G2** lineage, similar to the DTI strain, it is closer to the **G2a** strain. In contrast, DTI is more closely related to the China-like **G2b** strain. Since the discovery of an exotic strain in 2015, suggested to be a recombinant between **G2a** and **G2b**, the exotic strains identified in Thailand have been associated with **G2b**. The **G2a** endemic strain in Thailand remains similar to the strain discovered during the large-scale outbreak, primarily consisting of **TH1-G2a**, with or without the unique insertion (INS) (N > T)REY at position 233, as previously reported [9].

The recombinant strain identified in 2015 (accession number MZ090589) also contains a unique insertion, TTGR, at position 234, which is very close to the insertion found in **TH1-G2a** INS. Interestingly, this recombinant might suggest that the **G2b** strain, which may be the ancestor of the novel strain identified here, might have been introduced before the discovery of CBR/2014 and BP-2016. This is because the virus requires time to undergo substitution and recombination events and to adapt to infect its host before the novel strain emerges. Additionally, similar insertion regions have been observed in some Mexican strains (accession numbers KY828997, KY828998, MH004413, and MH004414), which feature an insertion of (A > L)GLA at amino acid position 232. Epitope predictions from the VCF (Tables S5 and S6) indicate that these changes affect both linear and discontinuous epitopes (DE5). Structural predictions suggest that this region is located outside the S1 subunit (Figure 4 and Table S6). This implies that this region may play a significant role in PEDV infection, particularly in swine herds in Thailand and Mexico.

For the novel DTI strain, a unique insertion was identified at position 306, which includes the sequence EDLKS. This insertion is located in a distinct region compared to previously described strains. Notably, it lies outside the S1 subunit but slightly above the region found in previously described strains such as **TH1-G2a INS** (VCF; Figure 4 and Table S6).

The EDLKS sequence is reminiscent of the amino acid change observed in certain South Korean strains (MH052684 and MN971595), which possess a similar amino acid sequence (EDLKS) at position 1298, located within the heptad repeat region 2 (HR2). Although the function of these amino acids remains unclear, DTI1 exhibits altered epitopes in a pattern similar to VCF. Specifically, DTI1 shows a loss of epitopes between positions 31 to 32 and 42 to 46 when compared to BP-2016, CBR1, and NPPED (Table S5). Moreover, the inserted amino acids appear to create a novel epitope (DE8; Tables S5 and S6) due to their unique insertion, which has not been observed in other strains. This novel epitope may play an important role in either host–cell attachment or immune evasion.

In a previous report [13], the epitope mAbA3 (amino acid sequence EGFSFNNWFLLS at positions 252–263) was identified as a novel neutralizing epitope in Thailand **G2**-PEDV. However, in our study, this epitope was not predicted by the software, and this site remained a conserved region in our sequences. This novel epitope is located within the S1 structure, beneath DE6 and DE8, as predicted in the epitopes for DTI described in our study (Table S6).

Since PEDV lacks an effective vaccine, particularly in high-variation regions like Southeast Asia, planned exposure is a more suitable approach for herd health management. However, the results of this study suggest that planned exposure using the predominant **G2a** strain is less effective against **G2b**. Additionally, based on the authors’ experience, we observed a shorter interval between outbreaks in areas where **G2b** was discovered. This may indicate that controlling PEDV using planned exposure has been unsuccessful in these cases. According to discussions with the veterinarian from Farm A (VCF-affected), less than half of the piglets died, while most piglets from Farm B (DTI-affected) perished. Notably, piglets at Farm A died earlier (approximately 1–3 days post-farrowing) compared to those at Farm B, where most deaths occurred after 3 days post-farrowing. Although disease progression after 3 days post-farrowing was not observed at Farm A, these data were not included in our analysis as they fell outside the scope of the observed information. Nonetheless, these findings suggest that the DTI strain may have a greater ability to persist in the host longer than the VCF strain, potentially causing higher mortality rates, despite the VCF-affected herds having immunization against **G2a** through planned exposure.

From this study and the surveillance studies conducted by our laboratory, **G2a**, particularly with the unique insertion, remains the predominant strain in Thailand. However, in some areas, **G2b** has overtaken **G2a**, especially in farms previously affected by **G2a**, notably in the central and eastern regions with high pig populations, except in Ratchaburi province. Our findings highlight the need for caution in preventive strategies in Thailand, particularly in avoiding the use of different clusters for protection against PEDV, such as using **G2a** to protect against **G2b**, although they are the same genogroup. Further studies should explore the effects of using strains within the same cluster but with point mutations at the insertion site, similar to **TH1-G2a** and **TH1-G2a INS**.

To mitigate issues with planned exposure, surveillance studies in high-risk areas should be conducted to identify strains like **G2a** or **G2b**. Our laboratory continues to use the **TH1-6** lineage for surveillance of PEDV in Thailand, making it easier to identify any exotic strains and determine their origins.

## 5. Conclusions

We discovered a novel strain, DTI, classified as **G2b** or TH4 based on our lineage classification. This strain is very similar to the China PEDV but contains a unique insertion that might act as a new epitope suggested by the software prediction, potentially serving as a decoy for immune evasion. Although DTI tends to show fewer clinical signs compared to the predominant strain in Thailand, the mortality rate might be higher in herds using planned exposure with **G2a**-affected intestines. This is indicated by the degree of severity of piglet villi, despite no significant difference in immunization levels against the endemic strain like **TH1-G2a INS**. These findings raise concerns about the impact of the external introduction of exotic strains to stable herds and provide insights for future phylogenetic studies of PEDV to clarify the relationship between them, especially the classification system, since Thailand is a source of **G2a**-PEDV.

## Data Availability

The DTI1-4 sequences from this study have been deposited in GENBANK^®^ under the accession numbers MW805354, MW805355, MW805356, and MW805357. The protein structures, along with epitope demonstrations of DTI1 and VCF, have been deposited on the 3D model website Sketchfab. They are accessible via the following URLs: DTI: https://sketchfab.com/3d-models/dti1-epitope-a4aadfe0bb9d4a81b1c48aff5c934baa and VCF: https://sketchfab.com/3d-models/vcf-epitope-56d9768ac1c44aeb99d0d0e8c10a887f (accessed on 7 January 2025).

## Supplementary Materials

The following supporting information can be downloaded at https://www.mdpi.com/article/10.3390/ani15020225/s1, Table S1: Primers used in this study [15,17,18]; Table S2: The accession numbers of the representative sequences in this study; Table S3: Parities (Par), viral neutralization (VN) titers, and the S/P ratio of immunoglobulin A (IgA) and G (IgG) at 0 DPF and 3 DPF in Farms A and B in this study; Table S4: The villous length (µM) and villous-height-to-crypt-depth ratio (VCR) of the duodenum, jejunum, and ileum in piglet intestinal samples; Table S5: Linear epitope prediction of the representative sequences used in this study. The highlighted cells indicate the predicted epitopes, while red text denotes amino acid differences compared to those of DTI1. The blue, purple, and green text in the position column denotes the COE and RBD domains (497th to 636th), SS2 (744th to 751st), and SS6 (761st to 767th), respectively. The right panel displays the prediction values (for complete data of Table S5, please see File S1). Table S6: Discontinuous epitope (DE) prediction of the representative sequences used in this study. The color highlights in each cell correspond to the structural model available at the deposit URL. The lines between rows indicate the positions and amino acid data representing the discontinuous residues. “NA” denotes unavailable data (information on the 3D structure of the strain in this study can be found in Files S2 and S3).

## References

[1] Pensaert M., De Bouck P. (1978). A new coronavirus-like particle associated with diarrhea in swine. Arch. Virol..

[2] Chasey D., Cartwright S. (1978). Virus-like particles associated with porcine epidemic diarrhoea. Res. Vet. Sci..

[3] Pospischil A., Stuedli A., Kiupel M. (2002). Update on porcine epidemic diarrhea. J. Swine Health Prod..

[4] Luo H., Liang Z., Lin J., Wang Y., Liu Y., Mei K., Zhao M., Huang S. (2024). Research progress of porcine epidemic diarrhea virus S protein. Front. Microbiol..

[5] Stevenson G.W., Hoang H., Schwartz K.J., Burrough E.R., Sun D., Madson D., Cooper V.L., Pillatzki A., Gauger P., Schmitt B.J. (2013). Emergence of Porcine epidemic diarrhea virus in the United States: Clinical signs, lesions, and viral genomic sequences. J. Vet. Diagn. Investig..

[6] Kocherhans R., Bridgen A., Ackermann M., Tobler K. (2001). Completion of the porcine epidemic diarrhoea coronavirus (PEDV) genome sequence. Virus Genes.

[7] Sun M., Ma J., Wang Y., Wang M., Song W., Zhang W., Lu C., Yao H. (2015). Genomic and epidemiological characteristics provide new insights into the phylogeographical and spatiotemporal spread of porcine epidemic diarrhea virus in Asia. J. Clin. Microbiol..

[8] Lee C. (2015). Porcine epidemic diarrhea virus: An emerging and re-emerging epizootic swine virus. Virol. J..

[9] Stott C.J., Temeeyasen G., Tripipat T., Kaewprommal P., Tantituvanont A., Piriyapongsa J., Nilubol D. (2017). Evolutionary and epidemiological analyses based on spike genes of porcine epidemic diarrhea virus circulating in Thailand in 2008–2015. Infect Genet. Evol..

[10] Yu L., Liu Y., Wang S., Zhang L., Liang P., Wang L., Dong J., Song C. (2020). Molecular Characteristics and Pathogenicity of Porcine Epidemic Diarrhea Virus Isolated in Some Areas of China in 2015–2018. Front Vet Sci.

[11] Chang S.H., Bae J.L., Kang T.J., Kim J., Chung G.H., Lim C.W., Laude H., Yang M.S., Jang Y.S. (2002). Identification of the epitope region capable of inducing neutralizing antibodies against the porcine epidemic diarrhea virus. Mol. Cells.

[12] Sun D., Feng L., Shi H., Chen J., Cui X., Chen H., Liu S., Tong Y., Wang Y., Tong G. (2008). Identification of two novel B cell epitopes on porcine epidemic diarrhea virus spike protein. Vet. Microbiol..

[13] Thavorasak T., Chulanetra M., Glab-Ampai K., Teeranitayatarn K., Songserm T., Yodsheewan R., Sae-Lim N., Lekcharoensuk P., Sookrung N., Chaicumpa W. (2022). Novel Neutralizing Epitope of PEDV S1 Protein Identified by IgM Monoclonal Antibody. Viruses.

[14] Kirchdoerfer R.N., Bhandari M., Martini O., Sewall L.M., Bangaru S., Yoon K.-J., Ward A.B. (2021). Structure and immune recognition of the porcine epidemic diarrhea virus spike protein. Structure.

[15] Temeeyasen G., Srijangwad A., Tripipat T., Tipsombatboon P., Piriyapongsa J., Phoolcharoen W., Chuanasa T., Tantituvanont A., Nilubol D. (2014). Genetic diversity of ORF3 and spike genes of porcine epidemic diarrhea virus in Thailand. Infect. Genet. Evol..

[16] Cheun-Arom T., Temeeyasen G., Tripipat T., Kaewprommal P., Piriyapongsa J., Sukrong S., Chongcharoen W., Tantituvanont A., Nilubol D. (2016). Full-length genome analysis of two genetically distinct variants of porcine epidemic diarrhea virus in Thailand. Infect. Genet. Evol..

[17] Pan Y., Tian X., Li W., Zhou Q., Wang D., Bi Y., Chen F., Song Y. (2012). Isolation and characterization of a variant porcine epidemic diarrhea virus in China. Virol. J..

[18] Zhao P.D., Tan C., Dong Y., Li Y., Shi X., Bai J., Jiang P. (2015). Genetic variation analyses of porcine epidemic diarrhea virus isolated in mid-eastern China from 2011 to 2013. Can. J. Vet. Res..

[19] Benson D.A., Cavanaugh M., Clark K., Karsch-Mizrachi I., Ostell J., Pruitt K.D., Sayers E.W. (2018). GenBank. Nucleic Acids Res..

[20] Katoh K., Misawa K., Kuma K., Miyata T. (2002). MAFFT: A novel method for rapid multiple sequence alignment based on fast Fourier transform. Nucleic Acids Res..

[21] Rambaut A. (2018). FigTree v1.4.4.

[22] Trifinopoulos J., Nguyen L.-T., von Haeseler A., Minh B.Q. (2016). W-IQ-TREE: A fast online phylogenetic tool for maximum likelihood analysis. Nucleic Acids Res..

[23] Rambaut A., Lam T.T., Max Carvalho L., Pybus O.G. (2016). Exploring the temporal structure of heterochronous sequences using TempEst (formerly Path-O-Gen). Virus Evol..

[24] Hall T.A. (1999). BioEdit: A user-friendly biological sequence alignment editor and analysis program for Windows 95/98/NT. Nucleic Acids Symposium Series.

[25] Srijangwad A., Stott C.J., Temeeyasen G., Senasuthum R., Chongcharoen W., Tantituvanont A., Nilubol D. (2017). Immune response of gilts to single and double infection with porcine epidemic diarrhea virus. Arch. Virol..

[26] Waterhouse A., Bertoni M., Bienert S., Studer G., Tauriello G., Gumienny R., Heer F.T., de Beer T.A.P., Rempfer C., Bordoli L. (2018). SWISS-MODEL: Homology modelling of protein structures and complexes. Nucleic Acids Res..

[27] Wimley W.C., White S.H. (1996). Experimentally determined hydrophobicity scale for proteins at membrane interfaces. Nat. Struct. Biol..

[28] Wang J., Youkharibache P., Zhang D., Lanczycki C.J., Geer R.C., Madej T., Phan L., Ward M., Lu S., Marchler G.H. (2020). iCn3D, a web-based 3D viewer for sharing 1D/2D/3D representations of biomolecular structures. Bioinformatics.

[29] Ponomarenko J., Bui H.H., Li W., Fusseder N., Bourne P.E., Sette A., Peters B. (2008). ElliPro: A new structure-based tool for the prediction of antibody epitopes. BMC Bioinform..

[30] Pettersen E.F., Goddard T.D., Huang C.C., Couch G.S., Greenblatt D.M., Meng E.C., Ferrin T.E. (2004). UCSF Chimera--a visualization system for exploratory research and analysis. J. Comput. Chem..

[31] R Core Team (2024). R: A Language and Environment for Statistical Computing.

[32] Huang C.Y., Draczkowski P., Wang Y.S., Chang C.Y., Chien Y.C., Cheng Y.H., Wu Y.M., Wang C.H., Chang Y.C., Chang Y.C. (2022). In situ structure and dynamics of an alphacoronavirus spike protein by cryo-ET and cryo-EM. Nat. Commun..

[33] Yang C., Sun J.-Y., Li X.-L., Cheng N., Wang K.-Y., Li L.-Q., Cheng X.-J., Sun Y.-F. (2024). Emerging and re-emerging genotype 2c porcine epidemic diarrhoea virus with high pathogenicity in China. J. Infect..

[34] Li L., Li B., Wang J., Liu L., Li Y., Sun S., Yin S., Zhang L., Liu X., Xu X. (2024). A novel recombination porcine epidemic diarrhea virus isolated from Gansu, China: Genetic characterization and pathogenicity. Vet. Microbiol..

[35] Jermsutjarit P., Mebumroong S., Watcharavongtip P., Lin H., Tantituvanont A., Kaeoket K., Piñeyro P., Nilubol D. (2024). Evolution and virulence of porcine epidemic diarrhea virus following in vitro and in vivo propagation. Sci. Rep..

[36] Yamagami T., Miyama T., Toyomaki H., Sekiguchi S., Sasaki Y., Sueyoshi M., Makita K. (2021). Analysis of the effect of feedback feeding on the farm-level occurrence of porcine epidemic diarrhea in Kagoshima and Miyazaki Prefectures, Japan. J. Vet. Med. Sci..

