# Supplementary material for: Detection and Phylogenetic Analysis of an Exotic Strain of Porcine Epidemic Diarrhea Virus and Its Effect on an Affected Herd Immunized Against the Endemic Strain in Thailand
Source: Animals (Basel). 2025 Jan 15;15(2):225. doi: 10.3390/ani15020225 (PMC11759134; doi:10.3390/ani15020225)
Supplement: Supplementary file 1 [file animals-15-00225-s001.zip › animals-3412620-supplementary.pdf]

animals

MDPI

Supplementary Materials

Table S1. Primers used in this study.

Primer Name	Sequence 5′ to 3′	Position	Product Size	References
1PEDV F	GCG TTC CGT CGC CTT CTA CA	186-205	2562	[17]
1PEDV R	CAG GAA TCT GGA AGA CAC TTG CA	2747-2725		[17]
2PEDV F	GTA TTA TGC CAC CAG TGT CCC A	2659-2680	2295	[17]
2PEDV R	CAG TTG CCA GCA GGC ACT GT	4953-4934		[17]
PEDV/3278/F	ACA GGA TCT GCT TGA TGT GG	3277-3296	1858	This study
PEDV/5115/R	CAC GCT AAG GTG GGA CAA CT	5134-5115		This study
PEDV/4587/F	TGT GTT GGT GAT CGC CAC TG	4568-4587	979	This study
PEDV/5527/R	CC TGT AGA CCT GCT GAC TTG	5546-5527		This study
PEDV/5114/F	TTG GTG ATG CCA CGT TGT ATT CC	5091-5113	2381	This study
3PEDV R	CAA TGT GCT CTT GCA ATC CTG CA	7471-7449		[17]
PEDV/6718/F	CAG TAT CTT AAC ATT CTT GGT GTG	6695-6718	1787	This study
PEDV/8462/R	CCA ACG CGA CAG TAG GTC AT	8481-8462		This study
4PEDV F	CTG TTA AGT TAG TGG ACT CAG CGT	7323-7346	1159	[17]
PEDV/8462/R	CCA ACG CGA CAG TAG GTC AT	8481-8462		This study
PEDV/8437/F	TATC TCA CGC GGC TTT GGC	8419-8437	1383	This study
PEDV/9782/R	CCA ACA TGA CAG CCT GAA CC	9801-9782		This study
PEDV/9543/F	CAT GCG AGG TGC TTT GTT GC	9523-9542	1925	This study
PEDV/11429/R	GCT GAG TAG AAG CCT CAC GG	11447-11428		This study
PEDV/11353/F	CGC CAA CAG TAT GAA GAT GC	11333-11352	1106	This study
PEDV/12420/R	GGA TGC TCA ACA TGT GCT CT	12438-12419		This study
6PEDV F	ACG CTT GCA GGC TGG TAA ACA	12178-12198	2261	[17]
6PEDV R	TGG GCA GTG CTC TAT CGC ACT	14438-14458		[17]
7PEDV F	ATA CTA GGG GCG CTT CGG TT	14318-14337	2459	[17]
7PEDV R	GTC AGG GTG CAC AGG AAT GAA	16776-16756		[17]
PEDV/16663/F	CGT TGT CAC TCA ACG CAT G	16644-16662	1089	This study
PEDV/17774/R	AAA CCA CCA GCC CAC AAC AC	17732-17713		This study
PEDV/17592/F	AGC TCG TGC TCC ACC AGG	17514-17591	1485	This study
8PEDV R	CCG ACT TTG AGG GTG ACG TC	18998-18976		[17]
9PEDV F	CGT AGC TTT TGA GTT GTA TGC CA	18870-18892	2466	[17]
9PEDV R	GCA ATT AGC TGT ACA GGG TTC A	21335-21314		[17]
PEDV S1 F	TAG TGA TGT TGT GTT AGG CTT GTT G	20535-20559	1197	[18]
PEDV S1 R	AGG ATC TGA GGA ATT ACT GCA AAC	21731-21690		[18]
PEDV S2 F	CAT ACT GCT TTA GGA ACA AAT CTT	21660-21683	1039	[18]
PEDV S2 R	ACA ACT GTC CAG AAT CAG ATG TAT A	22698-22674		[18]
PEDV S3 F	TTA TTA CCC TTA CAA ATT CTA GC	22633-22655	1083	[18]
PEDV S3 R	GAC TAA TAG CCT CTT TAA CAC TCT	23715-23692		[18]
PEDV S4 CJS F	TGT TCT ACA GCG GAA CCA GC	23618-23637	1189	This study
PEDV S4 CJS R	TCA CTG CAC GTG GAC CTT T	24806-24788		This study
PEDV ORF3 F	CCT AGA CTT CAA CCT TAC GA	24759-24778	774	[15]
PEDV ORF3 R	CAG GAA AAA GAG TAC GAA AA	25532-25513		[15]
PEDV/25343/F	TAT ACG TGG GCG GCA AGA AG	25321-25340	1420	This study
PEDV/26724/R	TCA GAC GCC TTT CTG ACA CC	26740-26721		This study

https://doi.org/10.3390/xxxxx

Primer Name	Sequence 5′ to 3′	Position	Product Size	References
12PEDV F	TCG TCC AAT TGG TTA ATC TGT GC	25552-25574	2306	[17]
12PEDV R	TAC CGT TGT GTG CAA GAC CAA	27857-27834		[17]
PEDV/26702/F	TTC TGG GTT GCT AAA GAA GGC G	26676-26697	-	This study
PEDV/2880/F	CAG CAC TCA ATG GTA GTA TTG C	2859-2880	987	This study
PEDV/3826/R	AC GGT TCC AAA GTT GGC GTC	3845-3826		This study
PEDV/5114/F	TTG GTG ATG CCA CGT TGT ATT CC	5091-5113	1691	This study
PEDV/6760/R	GTC GCC AAA AAC ATC AAA AGG C	6781-6760		This study
PEDV/19252/F	GAA GAA CGG CAA GTT CGA GG	19233-19252	1110	This study
PEDV/20322/R	AAT GCC TCT GAT GAT GAC GTG	20342-20322		This study

Table S2. The accession numbers of the representative sequences in this study.

Acc. No.	Country	Year	Acc. No.	Country	Year	Acc. No.	Country	Year
LT898433	AUS	2015	MZ364311	CHN	2017	MH052684	KOR	2017
AF353511	BEL	1978	MH061341	CHN	2018	MH243319	KOR	2018
LT905451	BEL	1978	MK138516	CHN	2018	MK032689	KOR	2018
KR003452	BEL	2015	MK796238	CHN	2018	MN971595	KOR	2019
KM189367	CAN	2014	MN594506	CHN	2018	MH006958	MEX	2013
JN547228	CHN	1986	MT166307	CHN	2018	MH004412	MEX	2016
MH726393	CHN	2011	MK140812	CHN	2018	MT490315	MEX	2018
KC196276	CHN	2011	MK140814	CHN	2018	MK558089	PHL	2017
JX524137	CHN	2011	ON068583	CHN	2018	MZ268115	POL	2015
MH726400	CHN	2011	MK606368	CHN	2018	MZ325486	POL	2016
JQ282909	CHN	2011	MN315264	CHN	2018	LT898435	ROM	2015
KM609203	CHN	2012	MZ364315	CHN	2019	KU297956	SLO	2015
ON262797	CHN	2012	MZ364316	CHN	2019	MN692780	SPA	2014
KC140102	CHN	2012	MN841671	CHN	2019	KC764953	THA	2008
KX580953	CHN	2013	MT303066	CHN	2019	KY000559	THA	2008
KP765609	CHN	2013	OL348059	CHN	2019	KY828922	THA	2008
KU664503	CHN	2013	OP529824	CHN	2019	KC764952	THA	2008
KM609210	CHN	2014	MT263014	CHN	2019	LC053455	THA	2010
MH726370	CHN	2014	OP326239	CHN	2019	KC764955	THA	2010
MH726380	CHN	2014	OQ269589	CHN	2020	KC764956	THA	2011
MH726374	CHN	2014	OL446966	CHN	2020	KC764957	THA	2011
MH056657	CHN	2014	OM393722	CHN	2020	KC764959	THA	2011
MH726408	CHN	2014	ON964511	CHN	2020	KF724937	THA	2011
KX064280	CHN	2014	MW762534	CHN	2020	KX981899	THA	2011
KX534205	CHN	2015	OK584017	CHN	2021	KC764954	THA	2012
KR095279	CHN	2015	OQ979198	CHN	2021	KC764958	THA	2012
KY007140	CHN	2015	OQ979201	CHN	2021	KC764960	THA	2012
KY420075	CHN	2015	OR707084	CHN	2021	KF724936	THA	2012
KY007139	CHN	2015	OL347994	CHN	2021	KF724935	THA	2012
KU847996	CHN	2015	OR234022	CHN	2021	KF724938	THA	2013
KX839248	CHN	2015	ON960076	CHN	2021	KR610993	THA	2014
KX839249	CHN	2015	OQ915150	CHN	2022	KR610994	THA	2014
KX839250	CHN	2015	OR061129	CHN	2022	KX981897	THA	2014
MZ364307	CHN	2016	OQ731918	CHN	2022	KX981898	THA	2014
MT338517	CHN	2016	OR123893	CHN	2022	KR610991	THA	2014
MT338518	CHN	2016	OQ378935	CHN	2022	KR610992	THA	2014
MZ380292	CHN	2016	OQ589489	CHN	2023	MZ090589	THA	2015
KX812524	CHN	2016	OR587951	CHN	2023	KX981900	THA	2015

Acc. No.	Country	Year	Acc. No.	Country	Year	Acc. No.	Country	Year
MG546689	CHN	2016	MK071624	COL	2014	LC496368	THA	2016
MF462814	CHN	2016	MK071629	COL	2015	MW805354	THA	2019
MK841494	CHN	2016	KR011756	FRA	2014	MW805355	THA	2019
MZ364310	CHN	2016	LT906582	GBR	1987	MW805356	THA	2019
MT547179	CHN	2017	OQ302146	HRV	2017	MW805357	THA	2019
MT547180	CHN	2017	KX289955	HUN	2016	MW165329	TWN	2013
MF807952	CHN	2017	KR061458	ITA	2009	MW165328	TWN	2014
MF807951	CHN	2017	KY111278	ITA	2016	KY929405	TWN	2015
MH726403	CHN	2017	LC063836	JPN	2013	KP403954	UKR	2014
MH726406	CHN	2017	LC063838	JPN	2014	KM975738	USA	2013
MH726383	CHN	2017	LC022792	JPN	2014	KU558702	USA	2013
MN037494	CHN	2017	GU937797	KOR	1998	KU893866	USA	2014
MK644601	CHN	2017	MF737355	KOR	2001	OR348434	USA	2014
ON075821	CHN	2017	JQ023161	KOR	2009	MG334555	USA	2017
MK862249	CHN	2017	MG781192	KOR	2014	KJ960178	VNM	2013
MH708243	CHN	2017	KY825241	KOR	2016	KT941120	VNM	2014
MH061338	CHN	2017	KY963963	KOR	2016	MT198679	VNM	2018
MK644603	CHN	2017	MH052689	KOR	2017			
MK644604	CHN	2017	MH052688	KOR	2017			

Table S3. Parities (Par), viral neutralization titers (VN), and the S/P ratios of immunoglobulin A (IgA) and immunoglobulin G (IgG) at 0 DPF and 3 DPF in Farms A and B in this study. a, b, c indicate significant differences.

Farm	Value	Par	0 DPF VN	3 DPF VN	0 DPF IgA	3 DPF IgA	0 DPF IgG	3 DPF IgG
**A**	Mean	3.10	3.40	2.70^{a}	1.506^{a}	0.471	0.658	0.341^{a}
(n=10)	SD	0.738	0.966	1.059	0.446	0.355	0.332	0.191
**B**	Mean	3.20	3.50	2.90^{b}	1.337^{a,b}	0.599	0.775	0.258^{a}
(n=10)	SD	0.422	0.972	0.876	0.335	0.501	0.345	0.125
**C**	Mean	3.05	3.45	3.00^{c}	1.450^{b}	0.500	0.700	0.300^{b}
(n=10)	SD	0.600	0.950	0.900	0.400	0.400	0.300	0.200
	P-value	0.924	0.183	0.032	0.053	0.344	0.149	0.003

Table S4. The villous length (μM) and villous-to-crypt depth ratio (VCR) of the duodenum, jejunum, and ileum in piglet intestinal samples. a, b, c indicate significant differences.

Farm	Value	Duodenum	Jejunum	Ileum	VCR duodenum	VCR jejunum	VCR ileum
**A**	Mean	363.811^{a}	250.854^{a}	222.521^{a}	0.829^{a}	0.584^{a}	0.871^{a}
(n=7)	SD	83.628	60.123	13.524	0.184	0.289	0.119
**B**	Mean	495.261^{a,b}	423.816^{b}	313.194^{a}	1.195^{a}	1.134^{a}	1.134^{a}
(n=8)	SD	139.532	105.954	85.384	0.139	0.324	0.308
**C**	Mean	495.261^{b}	423.816^{c}	313.194^{b}	1.195^{b}	1.134^{b}	1.134^{b}
(n=10)	SD	139.532	105.954	85.384	0.139	0.324	0.308
	P-value	**0.001**	**0.000**	**0.000**	**0.000**	**0.000**	**0.000**

Table S5. The linear epitope prediction of the representative sequences used in this study is shown. The highlighted cells indicate the predicted epitopes, while red text denotes amino acid differences compared to those of DTI1. The blue, purple, and green text in the position column denotes the COE & RBD domain (497th to 636th), SS2 (744th to 751st), and SS6 (761st to 767th), respectively. The right panel displays the prediction values (for complete data, please see File S1).

	Amino acids					
**Position**	**DTI1**	**BP-2016**	**CBR1**	**C9822**	**VCF**	**NPPED**
31	ASN	ASN	ASN	ASN	ASN	ASN
32	PHE	PHE	PHE	PHE	PHE	PHE
33	ARG	ARG	ARG	ARG	ARG	ARG
34	ARG	ARG	ARG	ARG	ARG	ARG
35	PHE	PHE	PHE	PHE	PHE	PHE
36	PHE	PHE	PHE	PHE	PHE	PHE
37	SER	SER	SER	SER	SER	SER
38	LYS	LYS	LYS	LYS	LYS	LYS
39	PHE	PHE	PHE	PHE	PHE	PHE
40	ASN	ASN	ASN	ASN	ASN	ASN
41	VAL	VAL	VAL	VAL	VAL	VAL
42	GLN	GLN	GLN	GLN	GLN	GLN
43	SER	**ALA**	**ALA**	**ALA**	**ALA**	**ALA**
44	PRO	PRO	PRO	PRO	PRO	PRO
45	ALA	ALA	ALA	ALA	ALA	ALA
46	VAL	VAL	VAL	VAL	VAL	VAL
47	VAL	VAL	VAL	VAL	VAL	VAL
48	VAL	VAL	VAL	VAL	VAL	VAL
49	LEU	LEU	LEU	LEU	LEU	LEU
50	GLY	GLY	GLY	GLY	GLY	GLY
51	GLY	GLY	GLY	GLY	GLY	GLY
52	TYR	TYR	TYR	TYR	TYR	TYR
53	LEU	LEU	LEU	LEU	LEU	LEU
54	PRO	PRO	PRO	PRO	PRO	PRO
55	THR	THR	**ILE**	**ILE**	**ILE**	**ILE**
56	GLY	GLY	GLY	GLY	GLY	GLY
57	GLU	GLU	GLU	GLU	GLU	GLU
58	ASN	ASN	ASN	ASN	ASN	ASN
59	GLN	GLN	GLN	GLN	GLN	GLN
60	GLY	GLY	GLY	GLY	GLY	GLY
61	VAL	VAL	VAL	VAL	VAL	VAL
62	ASN	ASN	ASN	ASN	ASN	ASN
63	SER	SER	SER	SER	SER	SER
64	THR	THR	THR	THR	THR	THR
65	TRP	TRP	TRP	TRP	TRP	TRP
66	TYR	TYR	TYR	TYR	TYR	TYR
67	CYS	CYS	CYS	CYS	CYS	CYS
68	ALA	ALA	ALA	ALA	ALA	ALA
69	GLY	GLY	GLY	GLY	GLY	GLY
70	GLN	GLN	GLN	GLN	GLN	GLN
71	HIS	HIS	HIS	HIS	HIS	HIS
72	PRO	**SER**	PRO	PRO	PRO	PRO
73	THR	THR	THR	THR	THR	THR

	Amino acids					
**Position**	**DTI1**	**BP-2016**	**CBR1**	**C9822**	**VCF**	**NPPED**
74	ALA	ALA	ALA	ALA	ALA	ALA
75	SER	SER	SER	SER	SER	SER
76	GLY	GLY	GLY	GLY	GLY	GLY
77	VAL	VAL	VAL	VAL	VAL	VAL
78	HIS	HIS	HIS	HIS	HIS	HIS
79	GLY	GLY	GLY	GLY	GLY	GLY
80	ILE	ILE	ILE	ILE	ILE	ILE
81	PHE	PHE	PHE	PHE	PHE	PHE
82	LEU	LEU	LEU	LEU	LEU	LEU
83	SER	SER	SER	SER	SER	SER
84	HIS	HIS	HIS	HIS	HIS	HIS
85	ILE	ILE	ILE	ILE	ILE	ILE
86	ARG	ARG	ARG	ARG	ARG	ARG
87	GLY	GLY	GLY	GLY	GLY	GLY
88	GLY	GLY	GLY	GLY	GLY	GLY
89	HIS	HIS	HIS	HIS	HIS	HIS
90	GLY	GLY	GLY	GLY	GLY	GLY
91	PHE	PHE	PHE	PHE	PHE	PHE
92	GLU	GLU	GLU	GLU	GLU	GLU
93	ILE	ILE	ILE	ILE	ILE	ILE
94	GLY	GLY	GLY	GLY	GLY	GLY
95	ILE	ILE	ILE	ILE	ILE	ILE
96	SER	SER	SER	SER	SER	SER
97	GLN	GLN	GLN	GLN	GLN	GLN
98	GLU	GLU	GLU	GLU	GLU	GLU
99	PRO	PRO	PRO	PRO	PRO	PRO
100	PHE	PHE	PHE	PHE	PHE	PHE
101	ASP	ASP	ASP	ASP	ASP	ASP
102	SER	SER	**PRO**	**PRO**	**PRO**	**PRO**
103	SER	SER	SER	SER	SER	SER
104	GLY	GLY	GLY	GLY	GLY	GLY
105	TYR	TYR	TYR	TYR	TYR	TYR
106	GLN	GLN	GLN	GLN	GLN	GLN
107	LEU	LEU	LEU	LEU	LEU	LEU
108	TYR	TYR	TYR	TYR	TYR	TYR
109	LEU	LEU	LEU	LEU	LEU	LEU
110	HIS	HIS	HIS	HIS	HIS	HIS
111	LYS	LYS	LYS	LYS	LYS	LYS
112	ALA	ALA	ALA	ALA	ALA	ALA
113	THR	THR	THR	THR	THR	THR
114	ASN	ASN	ASN	ASN	ASN	ASN
115	GLY	GLY	GLY	GLY	GLY	GLY
116	ASN	ASN	ASN	ASN	ASN	ASN
117	THR	THR	THR	THR	THR	THR
118	ASN	ASN	ASN	ASN	ASN	ASN
119	ALA	ALA	ALA	ALA	ALA	ALA
120	THR	THR	THR	THR	THR	THR
121	ALA	ALA	ALA	ALA	ALA	ALA
122	ARG	ARG	ARG	ARG	ARG	ARG

	Amino acids					
**Position**	**DTI1**	**BP-2016**	**CBR1**	**C9822**	**VCF**	**NPPED**
123	LEU	LEU	LEU	LEU	LEU	LEU
124	ARG	ARG	ARG	ARG	ARG	ARG
125	ILE	ILE	ILE	ILE	ILE	ILE
126	CYS	CYS	CYS	CYS	CYS	CYS
127	GLN	GLN	GLN	GLN	GLN	GLN
128	PHE	PHE	PHE	PHE	PHE	PHE
129	PRO	PRO	PRO	PRO	PRO	PRO
130	SER	SER	**ASP**	**ASN**	SER	SER
131	ILE	ILE	**ASN**	**ILE**	ILE	ILE
132	LYS	LYS	LYS	LYS	LYS	LYS
133	THR	THR	THR	THR	THR	THR
134	LEU	LEU	LEU	LEU	LEU	LEU
135	GLY	GLY	GLY	GLY	GLY	GLY
136	PRO	PRO	PRO	PRO	PRO	PRO
137	THR	THR	THR	THR	THR	THR
138	ALA	ALA	ALA	ALA	ALA	ALA
139	ASN	ASN	ASN	ASN	ASN	ASN
140	ASN	ASN	ASN	ASN	ASN	ASN
141	ASP	ASP	ASP	ASP	ASP	ASP
142	VAL	VAL	VAL	VAL	VAL	VAL
143	THR	THR	THR	THR	THR	THR
144	THR	THR	THR	THR	THR	THR
145	GLY	GLY	GLY	GLY	GLY	GLY
146	ARG	ARG	ARG	ARG	ARG	ARG
147	ASN	ASN	ASN	ASN	ASN	ASN
148	CYS	CYS	CYS	CYS	CYS	CYS
149	LEU	LEU	LEU	LEU	LEU	LEU
150	PHE	PHE	PHE	PHE	PHE	PHE
151	ASN	ASN	ASN	ASN	ASN	ASN
152	LYS	LYS	LYS	LYS	LYS	LYS
153	ALA	ALA	ALA	ALA	ALA	ALA
154	ILE	ILE	ILE	ILE	ILE	ILE
155	PRO	PRO	PRO	PRO	PRO	PRO
156	ALA	ALA	ALA	ALA	ALA	ALA
157	HIS	HIS	HIS	HIS	**TYR**	HIS
158	MET	MET	MET	MET	MET	MET
159	SER	SER	SER	SER	SER	SER
160	GLU	GLU	GLU	GLU	GLU	GLU
161	HIS	HIS	HIS	HIS	HIS	HIS
162	SER	SER	SER	SER	SER	SER
163	VAL	VAL	VAL	VAL	VAL	VAL
164	VAL	VAL	VAL	VAL	VAL	VAL
165	GLY	GLY	GLY	SER	GLY	GLY
166	ILE	ILE	ILE	ILE	ILE	ILE
167	THR	THR	THR	THR	THR	THR
168	TRP	TRP	TRP	TRP	TRP	TRP
169	ASP	ASP	ASP	ASP	ASP	ASP
170	ASN	ASN	ASN	ASN	ASN	ASN
171	ASP	ASP	ASP	ASP	ASP	ASP

	Amino acids					
**Position**	**DTI1**	**BP-2016**	**CBR1**	**C9822**	**VCF**	**NPPED**
172	ARG	ARG	ARG	ARG	**LEU**	ARG
173	VAL	VAL	VAL	GLY	VAL	VAL
174	THR	THR	THR	THR	**ALA**	THR
175	VAL	VAL	VAL	VAL	VAL	VAL
176	PHE	PHE	PHE	PHE	PHE	PHE
177	SER	SER	SER	SER	SER	SER
178	ASP	ASP	ASP	ASP	ASP	ASP
179	LYS	LYS	LYS	LYS	LYS	LYS
180	ILE	ILE	ILE	ILE	ILE	ILE
181	TYR	TYR	TYR	TYR	TYR	TYR
182	HIS	HIS	HIS	HIS	HIS	**TYR**
183	PHE	PHE	PHE	PHE	PHE	PHE
184	TYR	TYR	TYR	TYR	TYR	TYR
185	PHE	PHE	PHE	PHE	PHE	PHE
186	LYS	LYS	LYS	LYS	LYS	LYS
187	ASN	ASN	ASN	ASN	ASN	ASN
188	ASP	ASP	ASP	ASP	ASP	ASP
189	TRP	TRP	TRP	TRP	TRP	TRP
190	SER	SER	SER	SER	SER	SER
191	ARG	ARG	ARG	ARG	**HIS**	ARG
192	VAL	VAL	VAL	VAL	VAL	VAL
193	ALA	ALA	ALA	ALA	ALA	ALA
194	THR	THR	THR	THR	THR	THR
195	LYS	LYS	LYS	LYS	LYS	LYS
196	CYS	CYS	CYS	CYS	CYS	CYS
197	TYR	TYR	TYR	TYR	TYR	TYR
198	ASN	ASN	ASN	ASN	ASN	ASN
199	SER	SER	SER	SER	SER	SER
200	GLY	GLY	GLY	GLY	GLY	GLY
201	GLY	GLY	GLY	GLY	GLY	GLY
202	CYS	CYS	CYS	CYS	CYS	CYS
203	ALA	ALA	ALA	ALA	ALA	ALA
204	MET	MET	MET	MET	MET	MET
205	GLN	GLN	GLN	GLN	GLN	GLN
206	TYR	TYR	TYR	TYR	TYR	TYR
207	VAL	VAL	VAL	VAL	VAL	VAL
208	TYR	TYR	TYR	TYR	TYR	TYR
209	GLU	GLU	GLU	GLU	GLU	GLU
210	PRO	PRO	PRO	PRO	PRO	PRO
211	THR	THR	THR	THR	THR	THR
212	TYR	TYR	TYR	TYR	TYR	TYR
213	TYR	TYR	TYR	TYR	TYR	TYR
214	MET	MET	MET	MET	MET	MET
215	LEU	LEU	LEU	LEU	LEU	LEU
216	ASN	ASN	ASN	ASN	ASN	ASN
217	VAL	VAL	VAL	VAL	VAL	VAL
218	THR	THR	THR	THR	THR	THR
219	SER	SER	SER	SER	SER	SER
220	ALA	ALA	ALA	ALA	ALA	ALA

	Amino acids					
**Position**	**DTI1**	**BP-2016**	**CBR1**	**C9822**	**VCF**	**NPPED**
221	GLY	GLY	GLY	GLY	GLY	GLY
222	GLU	GLU	**LYS**	**GLU**	GLU	GLU
223	ASP	ASP	ASP	ASP	**ALA**	ASP
224	GLY	GLY	GLY	GLY	GLY	GLY
225	ILE	ILE	ILE	ILE	ILE	ILE
226	SER	SER	SER	SER	SER	SER
227	TYR	TYR	TYR	TYR	TYR	TYR
228	GLN	GLN	GLN	GLN	GLN	GLN
229	PRO	PRO	PRO	PRO	PRO	PRO
230	CYS	CYS	CYS	CYS	CYS	CYS
231	THR	THR	THR	THR	THR	THR
232	ALA	ALA	ALA	ALA	ALA	ALA
233	ASN	ASN	ASN	ASN	**THR**	ASN
233+1				THR	**ARG**	
233+2				THR	**GLU**	
233+3				GLY	**TYR**	
233+4				ARG		
234	CYS	CYS	CYS	CYS	CYS	CYS
235	ILE	ILE	ILE	ILE	ILE	ILE
236	GLY	GLY	GLY	GLY	GLY	GLY
237	TYR	TYR	TYR	TYR	TYR	TYR
238	ALA	ALA	ALA	ALA	ALA	ALA
239	ALA	ALA	ALA	ALA	ALA	ALA
240	ASN	ASN	ASN	ASN	ASN	ASN
241	VAL	VAL	VAL	VAL	VAL	VAL
242	PHE	PHE	PHE	PHE	PHE	PHE
243	ALA	ALA	ALA	ALA	ALA	ALA
244	THR	THR	THR	THR	THR	THR
245	GLU	GLU	GLU	GLU	GLU	GLU
246	PRO	PRO	PRO	PRO	PRO	PRO
247	ASN	ASN	ASN	ASN	ASN	ASN
248	GLY	GLY	GLY	GLY	GLY	GLY
249	HIS	HIS	HIS	HIS	HIS	HIS
250	ILE	ILE	ILE	ILE	ILE	ILE
251	PRO	PRO	PRO	PRO	PRO	PRO
252	GLU	GLU	GLU	GLU	GLU	GLU
253	GLY	GLY	GLY	GLY	GLY	GLY
254	PHE	PHE	PHE	PHE	PHE	PHE
255	SER	SER	SER	SER	SER	SER
256	PHE	PHE	PHE	PHE	PHE	PHE
257	ASN	ASN	ASN	ASN	ASN	ASN
258	ASN	ASN	ASN	ASN	ASN	ASN
259	TRP	TRP	TRP	TRP	TRP	TRP
260	PHE	PHE	PHE	PHE	PHE	PHE
261	LEU	LEU	LEU	LEU	LEU	LEU
262	LEU	LEU	LEU	LEU	LEU	LEU
263	SER	SER	SER	SER	SER	SER
264	ASN	ASN	ASN	ASN	ASN	ASN
265	ASP	ASP	ASP	ASP	ASP	ASP

	Amino acids					
**Position**	**DTI1**	**BP-2016**	**CBR1**	**C9822**	**VCF**	**NPPED**
266	SER	SER	SER	SER	SER	SER
267	THR	THR	THR	THR	THR	THR
268	VAL	VAL	**LEU**	**LEU**	**LEU**	**LEU**
269	LEU	LEU	**VAL**	**VAL**	**VAL**	**VAL**
270	HIS	HIS	HIS	HIS	HIS	HIS
271	GLY	GLY	GLY	GLY	GLY	GLY
272	LYS	LYS	LYS	LYS	LYS	LYS
273	VAL	VAL	VAL	VAL	VAL	VAL
274	VAL	VAL	VAL	VAL	VAL	VAL
275	SER	SER	SER	SER	SER	SER
276	ASN	ASN	ASN	ASN	ASN	ASN
277	GLN	GLN	GLN	GLN	GLN	GLN
278	PRO	PRO	PRO	PRO	PRO	PRO
279	LEU	LEU	LEU	LEU	LEU	LEU
280	LEU	LEU	LEU	LEU	LEU	LEU
281	VAL	VAL	VAL	VAL	VAL	VAL
282	ASN	ASN	ASN	ASN	ASN	ASN
283	CYS	CYS	CYS	CYS	CYS	CYS
284	LEU	LEU	LEU	LEU	LEU	LEU
285	LEU	LEU	LEU	LEU	LEU	LEU
286	ALA	ALA	ALA	ALA	ALA	ALA
287	ILE	ILE	**MET**	**ILE**	ILE	ILE
288	PRO	PRO	PRO	PRO	PRO	PRO
289	LYS	LYS	LYS	LYS	LYS	LYS
290	ILE	ILE	ILE	ILE	ILE	ILE
291	TYR	TYR	TYR	TYR	TYR	TYR
292	GLY	GLY	GLY	GLY	GLY	GLY
293	LEU	LEU	LEU	LEU	LEU	LEU
294	GLY	GLY	GLY	GLY	GLY	GLY
295	GLN	GLN	GLN	GLN	GLN	GLN
296	PHE	PHE	PHE	PHE	PHE	PHE
297	PHE	PHE	PHE	PHE	PHE	PHE
298	SER	SER	SER	SER	SER	SER
299	PHE	PHE	PHE	PHE	PHE	PHE
300	ASN	ASN	ASN	ASN	ASN	ASN
301	GLN	GLN	GLN	GLN	GLN	GLN
302	THR	THR	THR	SER	THR	THR
303	MET	MET	**ILE**	**ILE**	**ILE**	**ILE**
304	ASP	ASP	ASP	ASP	ASP	ASP
305	GLY	GLY	GLY	GLY	GLY	GLY
306+1	GLU					
306+2	ASP					
306+3	LEU					
306+4	LYS					
306+5	SER					
306	PHE	**VAL**	**VAL**	**VAL**	**VAL**	**VAL**
307	CYS	CYS	CYS	CYS	CYS	CYS
308	ASN	ASN	ASN	ASN	ASN	ASN
309	GLY	GLY	GLY	GLY	GLY	GLY

	Amino acids					
**Position**	**DTI1**	**BP-2016**	**CBR1**	**C9822**	**VCF**	**NPPED**
310	ALA	ALA	ALA	ALA	ALA	ALA
311	ALA	ALA	ALA	ALA	ALA	ALA
312	ALA	ALA	**VAL**	**VAL**	**VAL**	**VAL**
313	GLN	GLN	GLN	GLN	ARG	GLN
314	ARG	ARG	ARG	ARG	ARG	ARG
315	ALA	ALA	ALA	ALA	ALA	ALA
316	PRO	PRO	PRO	PRO	PRO	PRO
317	GLU	GLU	GLU	GLU	GLU	GLU
318	ALA	ALA	ALA	ALA	ALA	ALA
319	LEU	LEU	LEU	LEU	LEU	LEU
320	ARG	ARG	ARG	LYS	ARG	ARG
321	PHE	PHE	PHE	PHE	PHE	PHE
322	ASN	ASN	ASN	ASN	ASN	ASN
323	ILE	ILE	ILE	ILE	ILE	ILE
324	ASN	ASN	**ASP**	**ASP**	ASN	**ASP**
325	ASP	ASP	ASP	ASP	ASP	ASP
326	THR	THR	THR	THR	THR	THR
327	SER	SER	**ALA**	**SER**	SER	SER
328	VAL	VAL	VAL	VAL	VAL	VAL
329	ILE	ILE	ILE	ILE	ILE	ILE
330	LEU	LEU	LEU	LEU	LEU	LEU
331	ALA	ALA	ALA	ALA	ALA	ALA
332	GLU	GLU	GLU	GLU	GLU	GLU
333	GLY	GLY	GLY	GLY	GLY	GLY
334	SER	SER	SER	SER	SER	SER
335	ILE	ILE	ILE	ILE	ILE	ILE
336	VAL	VAL	VAL	VAL	VAL	VAL
337	LEU	LEU	LEU	LEU	LEU	LEU
338	HIS	HIS	HIS	HIS	HIS	HIS
339	THR	THR	THR	THR	THR	THR
340	ALA	ALA	ALA	ALA	ALA	ALA
341	LEU	LEU	LEU	LEU	LEU	LEU
342	GLY	GLY	GLY	GLY	GLY	GLY
343	THR	THR	THR	THR	THR	THR
344	ASN	ASN	ASN	ASN	ASN	ASN
345	LEU	LEU	**PHE**	**PHE**	**PHE**	**PHE**
346	SER	SER	SER	SER	SER	SER
347	PHE	PHE	PHE	PHE	PHE	PHE
348	VAL	VAL	VAL	VAL	VAL	VAL
349	CYS	CYS	CYS	CYS	CYS	CYS
350	SER	SER	SER	SER	SER	SER
351	ASN	ASN	ASN	ASN	ASN	ASN
352	SER	SER	SER	SER	SER	SER
353	SER	SER	SER	SER	SER	SER
354	ASP	ASP	ASP	ASP	**ASN**	ASP
355	PRO	PRO	PRO	PRO	PRO	PRO
356	HIS	HIS	HIS	HIS	HIS	HIS
357	SER	SER	**LEU**	**SER**	**LEU**	**LEU**
358	ALA	ALA	**THR**	**ALA**	ALA	ALA

	Amino acids					
**Position**	**DTI1**	**BP-2016**	**CBR1**	**C9822**	**VCF**	**NPPED**
359	ILE	ILE	**THR**	**THR**	**THR**	**THR**
360	PHE	PHE	PHE	PHE	PHE	PHE
361	ALA	ALA	ALA	ALA	**PRO**	ALA
362	ILE	ILE	ILE	ILE	ILE	ILE
363	PRO	PRO	PRO	PRO	PRO	PRO
364	LEU	LEU	LEU	LEU	LEU	LEU
365	GLY	GLY	GLY	GLY	GLY	GLY
366	ALA	ALA	ALA	ALA	ALA	ALA
367	THR	THR	THR	THR	THR	THR
368	GLN	GLN	GLN	GLN	GLN	GLN
369	VAL	VAL	VAL	VAL	VAL	VAL
370	PRO	PRO	PRO	PRO	PRO	PRO
371	TYR	TYR	TYR	TYR	TYR	TYR
372	TYR	TYR	TYR	TYR	TYR	TYR
373	CYS	CYS	CYS	CYS	CYS	CYS
374	PHE	PHE	PHE	PHE	PHE	PHE
375	LEU	LEU	**PRO**	**LEU**	LEU	LEU
376	LYS	LYS	LYS	LYS	LYS	LYS
377	VAL	VAL	VAL	VAL	**GLY**	VAL
378	ASP	ASP	ASP	ASP	ASP	ASP
379	THR	THR	THR	THR	THR	THR
380	TYR	TYR	TYR	TYR	TYR	TYR
381	ASN	ASN	ASN	ASN	ASN	ASN
382	SER	SER	SER	SER	SER	SER
383	THR	THR	THR	SER	THR	THR
384	VAL	VAL	VAL	VAL	VAL	VAL
385	TYR	TYR	TYR	TYR	TYR	TYR
386	LYS	LYS	LYS	LYS	LYS	LYS
387	PHE	PHE	PHE	PHE	PHE	**LEU**
388	LEU	LEU	LEU	LEU	LEU	LEU
389	ALA	ALA	ALA	ALA	ALA	ALA
390	VAL	VAL	VAL	VAL	VAL	VAL
391	LEU	LEU	LEU	LEU	LEU	LEU
392	PRO	PRO	PRO	HIS	PRO	**HIS**
393	PRO	PRO	PRO	PRO	PRO	PRO
394	THR	THR	THR	THR	THR	THR
395	VAL	VAL	VAL	VAL	VAL	VAL
396	ARG	ARG	ARG	ARG	ARG	ARG
397	GLU	GLU	GLU	GLU	GLU	GLU
398	ILE	ILE	ILE	ILE	ILE	ILE
399	VAL	VAL	VAL	VAL	VAL	VAL
400	ILE	ILE	ILE	ILE	ILE	ILE
401	THR	THR	THR	THR	THR	THR
402	LYS	LYS	LYS	LYS	LYS	LYS
403	TYR	TYR	TYR	TYR	TYR	TYR
404	GLY	GLY	GLY	GLY	GLY	GLY
405	ASP	ASP	ASP	ASP	ASP	ASP
406	VAL	VAL	VAL	VAL	VAL	VAL
407	TYR	TYR	TYR	TYR	TYR	TYR

	Amino acids					
**Position**	**DTI1**	**BP-2016**	**CBR1**	**C9822**	**VCF**	**NPPED**
408	VAL	VAL	VAL	VAL	VAL	VAL
409	ASN	ASN	ASN	ASN	ASN	ASN
410	GLY	GLY	GLY	GLY	GLY	GLY
411	PHE	PHE	PHE	PHE	PHE	PHE
412	GLY	GLY	GLY	GLY	GLY	GLY
413	TYR	TYR	TYR	TYR	TYR	TYR
414	LEU	LEU	LEU	LEU	LEU	LEU
415	HIS	HIS	HIS	HIS	HIS	HIS
416	LEU	LEU	LEU	LEU	LEU	LEU
417	GLY	GLY	GLY	GLY	GLY	GLY
418	LEU	LEU	LEU	LEU	LEU	LEU
419	LEU	LEU	LEU	LEU	LEU	LEU
420	ASP	ASP	ASP	ASP	ASP	ASP
421	ALA	ALA	ALA	ALA	ALA	ALA
422	VAL	VAL	VAL	VAL	VAL	VAL
423	THR	THR	THR	THR	THR	THR
424	ILE	ILE	ILE	ILE	ILE	ILE
425	ASN	ASN	ASN	ASN	ASN	ASN
426	PHE	PHE	PHE	PHE	PHE	PHE
427	THR	THR	THR	THR	THR	THR
428	GLY	GLY	GLY	GLY	GLY	GLY
429	HIS	HIS	HIS	HIS	HIS	HIS
430	GLY	**ARG**	GLY	GLY	GLY	GLY
431	ILE	**THR**	**THR**	**THR**	**THR**	**THR**
432	ASP	ASP	ASP	ASP	ASP	ASP
433	GLY	GLY	GLY	ASP	**ASP**	**ASP**
434	ASP	ASP	ASP	ASP	ASP	ASP
435	VAL	VAL	VAL	VAL	VAL	VAL
436	SER	SER	SER	SER	SER	SER
437	GLY	GLY	GLY	GLY	GLY	GLY
438	PHE	PHE	PHE	PHE	PHE	PHE
439	TRP	TRP	TRP	TRP	TRP	TRP
440	THR	THR	THR	THR	THR	THR
441	ILE	ILE	ILE	ILE	ILE	ILE
442	ALA	ALA	ALA	ALA	ALA	ALA
443	SER	SER	SER	SER	SER	SER
444	THR	THR	THR	THR	THR	THR
445	ASN	ASN	ASN	ASN	ASN	ASN
446	PHE	PHE	PHE	PHE	PHE	PHE
447	VAL	VAL	VAL	VAL	VAL	VAL
448	ASP	ASP	ASP	ASP	ASP	ASP
449	ALA	ALA	ALA	ALA	ALA	ALA
450	LEU	LEU	LEU	LEU	LEU	LEU
451	ILE	ILE	ILE	ILE	ILE	ILE
452	GLU	GLU	GLU	GLU	GLU	GLU
453	VAL	VAL	VAL	VAL	VAL	VAL
454	GLN	GLN	GLN	GLN	GLN	GLN
455	GLY	GLY	GLY	GLY	GLY	GLY
456	THR	THR	THR	THR	THR	THR

	Amino acids					
Position	DTI1	BP-2016	CBR1	C9822	VCF	NPPED
457	ALA	ALA	ALA	ALA	ALA	ALA
458	ILE	ILE	ILE	ILE	ILE	ILE
459	GLN	GLN	GLN	GLN	GLN	GLN
460	ARG	ARG	ARG	ARG	ARG	ARG
461	ILE	ILE	ILE	ILE	ILE	ILE
462	LEU	LEU	LEU	LEU	LEU	LEU
463	TYR	TYR	TYR	TYR	TYR	TYR
464	CYS	CYS	CYS	CYS	CYS	CYS
465	ASP	ASP	ASP	ASP	ASP	ASP
466	ASP	ASP	ASP	ASP	ASP	ASP
467	PRO	PRO	PRO	PRO	PRO	PRO
468	VAL	VAL	VAL	VAL	VAL	VAL
469	SER	SER	SER	SER	SER	SER
470	GLN	GLN	GLN	GLN	GLN	GLN
471	LEU	LEU	LEU	LEU	LEU	LEU
472	LYS	LYS	LYS	LYS	LYS	LYS
473	CYS	CYS	CYS	CYS	CYS	CYS
474	SER	SER	SER	SER	SER	SER
475	GLN	GLN	GLN	GLN	GLN	GLN
476	VAL	VAL	VAL	VAL	VAL	VAL
477	ALA	ALA	ALA	ALA	ALA	ALA
478	PHE	PHE	PHE	PHE	PHE	PHE
479	ASP	ASP	ASP	ASP	ASP	ASP
480	LEU	LEU	LEU	LEU	LEU	LEU
481	ASP	ASP	ASP	ASP	ASP	ASP
482	ASP	ASP	ASP	ASP	ASP	ASP
483	GLY	GLY	GLY	GLY	GLY	GLY
484	PHE	PHE	PHE	PHE	PHE	PHE
485	TYR	TYR	TYR	TYR	TYR	TYR
486	PRO	PRO	PRO	PRO	PRO	PRO
487	ILE	ILE	ILE	ILE	ILE	ILE
488	SER	SER	SER	SER	SER	SER
489	SER	SER	SER	SER	SER	SER
490	THR	**ILE**	THR	ARG	**ARG**	**ARG**
491	ASN	ASN	ASN	ASN	ASN	ASN
492	LEU	LEU	LEU	HIS	LEU	LEU
493	LEU	LEU	LEU	LEU	LEU	LEU
494	SER	SER	SER	SER	SER	SER
495	HIS	HIS	HIS	HIS	HIS	HIS
496	GLU	GLU	GLU	GLU	GLU	GLU
**497**	GLN	GLN	GLN	GLN	GLN	GLN
**498**	PRO	**SER**	PRO	PRO	PRO	PRO
**499**	THR	THR	THR	ILE	**ILE**	**ILE**
**500**	SER	SER	SER	SER	SER	SER
**501**	PHE	PHE	PHE	PHE	PHE	PHE
**502**	VAL	VAL	VAL	VAL	VAL	VAL
**503**	THR	THR	THR	THR	THR	THR
**504**	LEU	LEU	LEU	LEU	LEU	LEU
**505**	PRO	PRO	PRO	PRO	PRO	PRO

	Amino acids					
**Position**	**DTI1**	**BP-2016**	**CBR1**	**C9822**	**VCF**	**NPPED**
**506**	SER	SER	SER	SER	SER	SER
**507**	PHE	PHE	PHE	PHE	PHE	PHE
**508**	ASN	ASN	ASN	ASN	ASN	ASN
**509**	ASP	ASP	ASP	ASP	ASP	ASP
**510**	HIS	HIS	HIS	HIS	HIS	HIS
**511**	SER	SER	SER	SER	SER	SER
**512**	PHE	PHE	PHE	PHE	PHE	PHE
**513**	VAL	VAL	VAL	VAL	VAL	VAL
**514**	ASN	ASN	ASN	ASN	ASN	ASN
**515**	ILE	ILE	ILE	ILE	ILE	ILE
**516**	THR	THR	THR	THR	THR	THR
**517**	VAL	VAL	VAL	VAL	VAL	VAL
**518**	SER	SER	SER	SER	SER	SER
**519**	ALA	ALA	ALA	ALA	ALA	ALA
**520**	ALA	ALA	ALA	SER	**SER**	**SER**
**521**	PHE	PHE	PHE	PHE	PHE	PHE
**522**	GLY	GLY	GLY	GLY	GLY	GLY
**523**	ASP	**GLY**	**GLY**	**GLY**	**GLY**	**GLY**
**524**	HIS	HIS	HIS	TYR	HIS	HIS
**525**	SER	SER	**ARG**	**SER**	SER	SER
**526**	GLY	GLY	GLY	GLY	GLY	GLY
**527**	ALA	ALA	ALA	ALA	ALA	ALA
**528**	ASN	ASN	ASN	ASN	ASN	ASN
**529**	LEU	LEU	LEU	LEU	LEU	LEU
**530**	ILE	ILE	ILE	ILE	ILE	ILE
**531**	ALA	ALA	ALA	ALA	ALA	ALA
**532**	SER	SER	SER	SER	SER	SER
**533**	ASP	ASP	ASP	ASP	ASP	ASP
**534**	THR	THR	THR	THR	THR	THR
**535**	THR	THR	THR	THR	THR	THR
**536**	ILE	ILE	ILE	ILE	ILE	ILE
**537**	ASN	ASN	ASN	ASN	ASN	ASN
**538**	GLY	GLY	GLY	GLY	GLY	GLY
**539**	PHE	PHE	PHE	PHE	PHE	PHE
**540**	SER	SER	SER	SER	SER	SER
**541**	SER	SER	SER	SER	SER	SER
**542**	PHE	PHE	PHE	PHE	PHE	PHE
**543**	CYS	CYS	**ARG**	**CYS**	CYS	CYS
**544**	VAL	VAL	VAL	VAL	VAL	VAL
**545**	ASP	ASP	ASP	ASP	ASP	ASP
**546**	THR	THR	THR	THR	THR	THR
**547**	ARG	ARG	ARG	ARG	ARG	ARG
**548**	GLN	GLN	GLN	GLN	GLN	GLN
**549**	PHE	PHE	PHE	PHE	PHE	PHE
**550**	THR	THR	THR	THR	THR	THR
**551**	ILE	ILE	ILE	ILE	ILE	ILE
**552**	THR	THR	**SER**	**SER**	**SER**	**SER**
**553**	LEU	LEU	**ARG**	**LEU**	LEU	LEU
**554**	PHE	PHE	PHE	PHE	PHE	PHE

	Amino acids					
**Position**	**DTI1**	**BP-2016**	**CBR1**	**C9822**	**VCF**	**NPPED**
**555**	TYR	TYR	TYR	TYR	TYR	**CYS**
**556**	ASN	ASN	ASN	ASN	ASN	ASN
**557**	VAL	VAL	VAL	VAL	VAL	VAL
**558**	THR	THR	**PRO**	**THR**	THR	THR
**559**	ASN	ASN	**THR**	**ASN**	ASN	ASN
**560**	SER	SER	SER	SER	SER	SER
**561**	TYR	TYR	TYR	TYR	TYR	TYR
**562**	GLY	GLY	GLY	GLY	GLY	GLY
**563**	TYR	TYR	TYR	TYR	TYR	TYR
**564**	VAL	VAL	**GLY**	**VAL**	VAL	VAL
**565**	SER	SER	SER	SER	SER	SER
**566**	LYS	LYS	LYS	LYS	LYS	LYS
**567**	SER	SER	SER	SER	SER	SER
**568**	GLN	GLN	GLN	SER	GLN	GLN
**569**	ASP	ASP	**GLY**	**ASP**	ASP	ASP
**570**	SER	SER	SER	SER	SER	SER
**571**	ASN	ASN	ASN	ASN	ASN	ASN
**572**	CYS	CYS	CYS	CYS	CYS	CYS
**573**	PRO	PRO	PRO	PRO	PRO	PRO
**574**	PHE	PHE	PHE	PHE	PHE	PHE
**575**	THR	THR	THR	THR	THR	THR
**576**	LEU	LEU	LEU	LEU	LEU	LEU
**577**	GLN	GLN	GLN	GLN	GLN	GLN
**578**	SER	SER	SER	SER	SER	SER
**579**	VAL	VAL	VAL	VAL	VAL	VAL
**580**	ASN	ASN	ASN	ASN	ASN	ASN
**581**	ASP	ASP	ASP	ASP	ASP	ASP
**582**	TYR	TYR	TYR	TYR	TYR	TYR
**583**	LEU	LEU	LEU	LEU	LEU	LEU
**584**	SER	SER	SER	SER	SER	SER
**585**	PHE	PHE	PHE	PHE	PHE	PHE
**586**	SER	SER	SER	SER	SER	SER
**587**	LYS	LYS	LYS	LYS	LYS	LYS
**588**	PHE	PHE	PHE	PHE	PHE	PHE
**589**	CYS	CYS	CYS	CYS	CYS	CYS
**590**	VAL	VAL	VAL	VAL	VAL	VAL
**591**	SER	SER	SER	SER	SER	SER
**592**	THR	THR	THR	THR	THR	THR
**593**	SER	SER	SER	SER	SER	SER
**594**	LEU	LEU	LEU	LEU	LEU	LEU
**595**	LEU	LEU	LEU	LEU	LEU	LEU
**596**	ALA	ALA	ALA	ALA	ALA	ALA
**597**	SER	SER	SER	SER	SER	SER
**598**	ALA	ALA	ALA	ALA	ALA	ALA
**599**	CYS	CYS	CYS	CYS	CYS	CYS
**600**	THR	THR	THR	THR	THR	THR
**601**	ILE	ILE	ILE	ILE	ILE	ILE
**602**	ASP	ASP	ASP	ASP	ASP	ASP
**603**	LEU	LEU	LEU	LEU	LEU	LEU

	Amino acids					
**Position**	**DTI1**	**BP-2016**	**CBR1**	**C9822**	**VCF**	**NPPED**
**604**	PHE	PHE	PHE	PHE	PHE	PHE
**605**	GLY	GLY	GLY	GLY	GLY	GLY
**606**	HIS	**TYR**	**TYR**	**TYR**	**TYR**	**TYR**
**607**	PRO	PRO	PRO	PRO	PRO	PRO
**608**	GLU	GLU	GLU	GLU	**ASP**	GLU
**609**	PHE	PHE	PHE	PHE	PHE	PHE
**610**	GLY	GLY	GLY	GLY	GLY	GLY
**611**	SER	SER	SER	SER	SER	SER
**612**	GLY	GLY	GLY	GLY	GLY	GLY
**613**	VAL	VAL	VAL	VAL	VAL	VAL
**614**	LYS	LYS	LYS	LYS	LYS	LYS
**615**	PHE	PHE	PHE	PHE	PHE	PHE
**616**	THR	THR	THR	THR	THR	THR
**617**	SER	SER	SER	SER	SER	SER
**618**	LEU	LEU	LEU	LEU	LEU	LEU
**619**	TYR	TYR	TYR	TYR	TYR	TYR
**620**	PHE	PHE	PHE	PHE	PHE	PHE
**621**	GLN	GLN	GLN	GLN	GLN	GLN
**622**	PHE	PHE	PHE	PHE	PHE	PHE
**623**	THR	THR	THR	THR	THR	THR
**624**	LYS	LYS	LYS	LYS	LYS	LYS
**625**	GLY	GLY	GLY	GLY	GLY	GLY
**626**	GLU	GLU	GLU	GLU	GLU	GLU
**627**	LEU	LEU	LEU	LEU	LEU	LEU
**628**	ILE	ILE	ILE	ILE	ILE	ILE
**629**	THR	THR	THR	THR	THR	THR
**630**	SER	**GLY**	**GLY**	**GLY**	**GLY**	**GLY**
**631**	THR	THR	THR	THR	THR	THR
**632**	PRO	PRO	PRO	PRO	PRO	PRO
**633**	LYS	LYS	LYS	LYS	LYS	LYS
**634**	PRO	PRO	PRO	PRO	PRO	PRO
**635**	LEU	LEU	LEU	LEU	LEU	LEU
**636**	GLU	GLU	GLU	GLU	GLU	GLU
637	GLY	GLY	GLY	GLY	GLY	GLY
638	VAL	VAL	VAL	VAL	VAL	VAL
639	THR	THR	THR	THR	THR	THR
640	ASP	ASP	ASP	ASP	ASP	ASP
641	VAL	VAL	VAL	VAL	VAL	VAL
642	SER	SER	SER	SER	SER	SER
643	PHE	PHE	PHE	PHE	PHE	PHE
644	MET	MET	MET	MET	MET	MET
645	THR	THR	THR	THR	THR	THR
646	LEU	LEU	LEU	LEU	LEU	LEU
647	ASP	ASP	ASP	ASP	ASP	ASP
648	VAL	VAL	VAL	VAL	VAL	VAL
649	CYS	CYS	CYS	HIS	CYS	CYS
650	THR	THR	THR	THR	THR	THR
651	LYS	LYS	LYS	LYS	LYS	LYS
652	TYR	TYR	TYR	TYR	TYR	TYR

	Amino acids					
**Position**	**DTI1**	**BP-2016**	**CBR1**	**C9822**	**VCF**	**NPPED**
653	THR	THR	THR	THR	THR	THR
654	ILE	ILE	ILE	ILE	ILE	ILE
655	TYR	TYR	TYR	TYR	TYR	TYR
656	GLY	GLY	GLY	GLY	GLY	GLY
657	PHE	PHE	PHE	PHE	PHE	PHE
658	LYS	LYS	LYS	LYS	LYS	LYS
659	GLY	GLY	GLY	GLY	GLY	GLY
660	GLU	GLU	GLU	GLU	GLU	GLU
661	GLY	GLY	GLY	GLY	GLY	GLY
662	ILE	ILE	ILE	ILE	**VAL**	ILE
663	ILE	ILE	ILE	ILE	ILE	ILE
664	THR	THR	THR	THR	THR	THR
665	LEU	LEU	LEU	LEU	LEU	LEU
666	THR	THR	THR	THR	THR	THR
667	ASN	ASN	ASN	ASN	ASN	ASN
668	SER	SER	SER	SER	SER	SER
669	SER	SER	SER	SER	SER	SER
670	PHE	PHE	PHE	PHE	PHE	PHE
671	LEU	LEU	LEU	LEU	LEU	LEU
672	ALA	ALA	ALA	ALA	ALA	ALA
673	GLY	GLY	GLY	GLY	GLY	GLY
674	VAL	VAL	VAL	VAL	VAL	VAL
675	TYR	TYR	TYR	TYR	TYR	TYR
676	TYR	TYR	TYR	TYR	TYR	TYR
677	THR	THR	THR	THR	THR	THR
678	SER	SER	SER	SER	SER	SER
679	ASP	ASP	ASP	ASP	ASP	ASP
680	SER	SER	SER	SER	SER	SER
681	GLY	GLY	GLY	GLY	GLY	GLY
682	GLN	GLN	GLN	GLN	GLN	GLN
683	LEU	LEU	LEU	LEU	LEU	LEU
684	LEU	LEU	LEU	LEU	LEU	LEU
685	ALA	ALA	ALA	ALA	ALA	ALA
686	PHE	PHE	PHE	PHE	PHE	PHE
687	LYS	LYS	LYS	LYS	LYS	LYS
688	ASN	ASN	ASN	ASN	ASN	ASN
689	VAL	VAL	VAL	VAL	VAL	VAL
690	THR	THR	THR	THR	THR	THR
691	SER	SER	SER	SER	SER	SER
692	GLY	GLY	GLY	GLY	GLY	GLY
693	ALA	ALA	ALA	ALA	ALA	ALA
694	ILE	ILE	**VAL**	**VAL**	**VAL**	**VAL**
695	TYR	TYR	TYR	TYR	TYR	TYR
696	SER	SER	SER	SER	SER	SER
697	VAL	VAL	VAL	VAL	VAL	VAL
698	THR	THR	THR	THR	THR	THR
699	PRO	PRO	PRO	PRO	PRO	PRO
700	CYS	CYS	CYS	CYS	CYS	CYS
701	SER	SER	SER	SER	SER	SER

	Amino acids					
**Position**	**DTI1**	**BP-2016**	**CBR1**	**C9822**	**VCF**	**NPPED**
702	PHE	PHE	PHE	PHE	**TYR**	PHE
703	SER	SER	SER	SER	SER	SER
704	GLU	GLU	GLU	GLU	GLU	GLU
705	GLN	GLN	GLN	GLN	GLN	GLN
706	ALA	ALA	ALA	ALA	ALA	ALA
707	ALA	ALA	ALA	ALA	ALA	ALA
708	TYR	TYR	TYR	TYR	TYR	TYR
709	VAL	VAL	VAL	VAL	VAL	VAL
710	ASP	ASP	ASP	ASP	ASP	ASP
711	ASP	ASP	ASP	ASP	ASP	ASP
712	ASP	ASP	ASP	ASP	ASP	ASP
713	ILE	ILE	ILE	ILE	ILE	ILE
714	VAL	VAL	VAL	VAL	VAL	VAL
715	GLY	GLY	GLY	GLY	GLY	GLY
716	VAL	VAL	VAL	VAL	VAL	VAL
717	ILE	ILE	ILE	ILE	ILE	ILE
718	SER	SER	SER	SER	SER	SER
719	SER	SER	SER	SER	SER	SER
720	LEU	LEU	LEU	LEU	LEU	LEU
721	SER	SER	SER	SER	SER	SER
722	ASN	**SER**	**SER**	**ASN**	ASN	**SER**
723	SER	SER	SER	SER	SER	SER
724	THR	THR	THR	THR	THR	THR
725	PHE	PHE	PHE	PHE	PHE	PHE
726	ASN	ASN	ASN	ASN	ASN	ASN
727	ASN	ASN	**SER**	**SER**	**SER**	**SER**
728	THR	THR	THR	THR	THR	THR
729	ARG	ARG	ARG	ARG	ARG	ARG
730	GLU	GLU	GLU	GLU	GLU	GLU
731	LEU	LEU	LEU	LEU	LEU	LEU
732	PRO	PRO	PRO	PRO	PRO	PRO
733	GLY	GLY	GLY	GLY	GLY	GLY
734	PHE	PHE	PHE	PHE	PHE	PHE
735	PHE	PHE	PHE	PHE	PHE	PHE
736	TYR	TYR	TYR	TYR	TYR	TYR
737	HIS	HIS	HIS	HIS	HIS	HIS
738	SER	SER	SER	SER	SER	SER
739	ASN	ASN	ASN	ASN	ASN	ASN
740	ASP	ASP	ASP	ASP	ASP	ASP
741	VAL	**GLY**	**GLY**	**GLY**	**GLY**	**GLY**
742	SER	SER	SER	SER	SER	SER
743	ASN	ASN	ASN	ASN	ASN	ASN
**744**	CYS	CYS	CYS	CYS	CYS	CYS
**745**	THR	THR	THR	THR	THR	THR
**746**	GLU	GLU	GLU	GLU	GLU	GLU
**747**	PRO	PRO	PRO	PRO	PRO	PRO
**748**	VAL	VAL	VAL	VAL	VAL	VAL
**749**	LEU	LEU	LEU	LEU	LEU	LEU
**750**	VAL	VAL	VAL	VAL	VAL	VAL

	Amino acids					
**Position**	**DTI1**	**BP-2016**	**CBR1**	**C9822**	**VCF**	**NPPED**
**751**	TYR	TYR	TYR	TYR	TYR	TYR
752	SER	SER	SER	SER	SER	SER
753	ASN	ASN	ASN	ASN	ASN	ASN
754	ILE	ILE	ILE	ILE	ILE	ILE
755	GLY	GLY	GLY	GLY	GLY	GLY
756	VAL	VAL	VAL	VAL	VAL	VAL
757	CYS	CYS	CYS	CYS	CYS	CYS
758	LYS	LYS	LYS	LYS	LYS	LYS
759	SER	SER	SER	SER	SER	SER
760	GLY	GLY	GLY	GLY	GLY	GLY
**761**	SER	SER	SER	SER	SER	SER
**762**	ILE	ILE	ILE	ILE	ILE	ILE
**763**	GLY	GLY	GLY	GLY	GLY	GLY
**764**	TYR	TYR	TYR	TYR	TYR	TYR
**765**	VAL	VAL	VAL	VAL	VAL	VAL
**766**	SER	SER	**PRO**	**PRO**	**PRO**	**PRO**
**767**	SER	SER	SER	SER	SER	SER
768	GLN	GLN	GLN	GLN	GLN	GLN
769	SER	SER	SER	SER	SER	SER
770	GLY	GLY	GLY	GLY	GLY	GLY
771	GLN	GLN	GLN	GLN	GLN	GLN
772	VAL	VAL	VAL	VAL	VAL	VAL
773	LYS	LYS	LYS	LYS	LYS	LYS
774	ILE	ILE	ILE	ILE	ILE	ILE
775	ALA	ALA	ALA	ALA	ALA	ALA
776	PRO	PRO	PRO	PRO	PRO	PRO
777	THR	THR	THR	THR	THR	THR
778	VAL	VAL	VAL	VAL	VAL	VAL
779	THR	THR	THR	THR	THR	THR
780	GLY	GLY	GLY	GLY	GLY	GLY
781	ASN	ASN	ASN	ASN	ASN	ASN
782	ILE	ILE	ILE	ILE	ILE	ILE
783	SER	SER	SER	SER	SER	SER
784	ILE	ILE	ILE	ILE	ILE	ILE
785	PRO	PRO	PRO	PRO	PRO	PRO
786	THR	THR	THR	THR	THR	THR
787	ASN	ASN	ASN	ASN	ASN	ASN
788	PHE	PHE	PHE	PHE	PHE	PHE
789	SER	SER	SER	SER	SER	SER
790	MET	MET	MET	MET	MET	MET
791	SER	SER	SER	SER	SER	SER
792	ILE	ILE	ILE	ILE	ILE	ILE
793	ARG	ARG	ARG	ARG	ARG	ARG
794	THR	THR	THR	THR	THR	THR
795	GLU	GLU	GLU	GLU	GLU	GLU
796	TYR	TYR	TYR	TYR	TYR	TYR
797	LEU	LEU	LEU	LEU	LEU	LEU
798	GLN	GLN	GLN	GLN	GLN	GLN
799	LEU	LEU	LEU	LEU	LEU	LEU

	Amino acids					
**Position**	**DTI1**	**BP-2016**	**CBR1**	**C9822**	**VCF**	**NPPED**
800	TYR	TYR	TYR	TYR	TYR	TYR
801	ASN	ASN	ASN	ASN	ASN	ASN
802	THR	THR	THR	THR	THR	THR
803	PRO	PRO	PRO	PRO	PRO	PRO
804	VAL	VAL	VAL	VAL	VAL	VAL
805	SER	SER	SER	SER	SER	SER
806	VAL	VAL	VAL	VAL	VAL	VAL
807	ASP	ASP	ASP	ASP	ASP	ASP
808	CYS	CYS	CYS	CYS	CYS	CYS
809	ALA	ALA	ALA	ALA	ALA	ALA
810	THR	THR	THR	THR	THR	THR
811	TYR	TYR	TYR	TYR	TYR	TYR
812	VAL	VAL	VAL	VAL	VAL	VAL
813	CYS	CYS	CYS	CYS	CYS	CYS
814	ASN	ASN	ASN	ASN	ASN	ASN
815	GLY	GLY	GLY	GLY	GLY	GLY
816	ASN	ASN	ASN	ASN	ASN	ASN
817	SER	SER	SER	SER	SER	SER
818	ARG	ARG	ARG	ARG	ARG	ARG
819	CYS	CYS	CYS	CYS	CYS	CYS
820	LYS	LYS	LYS	LYS	LYS	LYS
821	GLN	GLN	GLN	GLN	GLN	GLN
822	LEU	LEU	LEU	LEU	LEU	LEU
823	LEU	LEU	LEU	LEU	LEU	LEU
824	THR	THR	THR	THR	THR	THR
825	GLN	GLN	GLN	GLN	GLN	GLN
826	TYR	TYR	TYR	TYR	TYR	TYR
827	THR	THR	THR	THR	THR	THR
828	ALA	ALA	ALA	ALA	ALA	ALA
829	ALA	ALA	ALA	ALA	ALA	ALA
830	CYS	CYS	CYS	CYS	CYS	CYS
831	LYS	LYS	LYS	LYS	LYS	LYS
832	THR	THR	THR	THR	THR	THR
833	ILE	ILE	ILE	ILE	ILE	ILE
834	GLU	GLU	GLU	GLU	GLU	GLU
835	SER	SER	SER	SER	SER	SER
836	ALA	ALA	ALA	ALA	ALA	ALA
837	LEU	LEU	LEU	LEU	LEU	LEU
838	GLN	GLN	GLN	GLN	GLN	GLN
839	LEU	LEU	LEU	LEU	LEU	LEU
840	SER	SER	SER	SER	SER	SER
841	ALA	ALA	ALA	ALA	ALA	ALA
842	ARG	ARG	ARG	ARG	ARG	ARG
843	LEU	LEU	**PRO**	**LEU**	LEU	LEU
844	GLU	GLU	GLU	GLU	GLU	GLU
845	SER	SER	SER	SER	SER	SER
846	ALA	ALA	ALA	VAL	**VAL**	**VAL**
847	GLU	GLU	GLU	GLU	GLU	GLU
848	VAL	VAL	VAL	VAL	VAL	VAL

	Amino acids					
**Position**	**DTI1**	**BP-2016**	**CBR1**	**C9822**	**VCF**	**NPPED**
849	ASN	ASN	ASN	ASN	ASN	ASN
850	SER	SER	SER	SER	SER	SER
851	MET	MET	MET	MET	MET	MET
852	LEU	LEU	LEU	LEU	LEU	LEU
853	THR	THR	THR	THR	THR	THR
854	ILE	ILE	ILE	ILE	ILE	ILE
855	SER	SER	SER	SER	SER	SER
856	GLU	GLU	GLU	GLU	GLU	GLU
857	GLU	GLU	GLU	GLU	GLU	GLU
858	ALA	ALA	ALA	ALA	ALA	ALA
859	LEU	LEU	LEU	LEU	LEU	LEU
860	GLN	GLN	GLN	GLN	GLN	GLN
861	LEU	LEU	LEU	LEU	LEU	LEU
862	ALA	ALA	ALA	ALA	ALA	ALA
863	THR	THR	THR	THR	THR	THR
864	ILE	ILE	ILE	ILE	ILE	ILE
865	SER	SER	SER	SER	SER	SER
866	SER	SER	SER	SER	SER	SER
867	PHE	PHE	PHE	PHE	PHE	PHE
868	ASN	ASN	ASN	ASN	ASN	ASN
869	GLY	GLY	GLY	GLY	GLY	GLY
870	ASP	ASP	ASP	ASP	ASP	ASP
871	GLY	GLY	GLY	GLY	GLY	GLY
872	TYR	TYR	TYR	TYR	TYR	TYR
873	ASN	ASN	ASN	ASN	ASN	ASN
874	PHE	PHE	PHE	PHE	PHE	PHE
875	THR	THR	THR	THR	THR	THR
876	ASN	ASN	ASN	ASN	ASN	ASN
877	VAL	VAL	VAL	VAL	VAL	VAL
878	LEU	LEU	LEU	LEU	LEU	LEU
879	GLY	GLY	GLY	GLY	GLY	GLY
880	VAL	VAL	VAL	VAL	VAL	VAL
881	SER	SER	SER	SER	SER	SER
882	VAL	VAL	VAL	VAL	VAL	VAL
883	TYR	TYR	TYR	TYR	TYR	TYR
884	ASP	ASP	ASP	ASP	**GLU**	**GLU**
885	PRO	PRO	PRO	PRO	PRO	PRO
886	ALA	ALA	ALA	ALA	ALA	ALA
887	SER	SER	SER	SER	SER	SER
888	ASP	**GLY**	**GLY**	**GLY**	**GLY**	**GLY**
889	ARG	ARG	ARG	ARG	ARG	ARG
890	VAL	VAL	VAL	VAL	VAL	VAL
891	VAL	VAL	VAL	VAL	VAL	VAL
892	GLN	GLN	GLN	HIS	**HIS**	**HIS**
893	LYS	LYS	LYS	LYS	LYS	LYS
894	ARG	ARG	ARG	ARG	ARG	ARG
895	SER	SER	SER	SER	SER	SER
896	PHE	PHE	PHE	PHE	PHE	PHE
897	ILE	ILE	ILE	ILE	ILE	ILE

	Amino acids					
Position	DTI1	BP-2016	CBR1	C9822	VCF	NPPED
898	GLU	GLU	GLU	GLU	GLU	GLU
899	ASP	ASP	ASP	ASP	ASP	ASP
900	LEU	LEU	LEU	LEU	LEU	LEU
901	LEU	LEU	LEU	LEU	LEU	LEU
902	PHE	PHE	PHE	PHE	PHE	PHE
903	ASN	ASN	ASN	ASN	ASN	ASN
904	LYS	LYS	LYS	LYS	LYS	LYS
905	VAL	VAL	VAL	VAL	VAL	VAL
906	VAL	VAL	VAL	VAL	VAL	VAL
907	THR	THR	THR	THR	THR	THR
908	ASN	ASN	ASN	ASN	ASN	ASN
909	GLY	GLY	GLY	GLY	GLY	GLY
910	LEU	LEU	LEU	LEU	LEU	LEU
911	GLY	GLY	GLY	GLY	GLY	GLY
912	THR	THR	THR	THR	THR	THR
913	VAL	VAL	VAL	VAL	VAL	VAL
914	ASP	ASP	ASP	ASP	ASP	ASP
915	GLU	GLU	GLU	GLU	GLU	GLU
916	ASP	ASP	ASP	ASP	ASP	ASP
917	TYR	TYR	TYR	TYR	TYR	TYR
918	LYS	LYS	LYS	LYS	LYS	LYS
919	ARG	ARG	ARG	ARG	ARG	ARG
920	CYS	CYS	CYS	CYS	CYS	CYS
921	SER	SER	SER	SER	SER	SER
922	ASN	ASN	ASN	ASN	ASN	ASN
923	GLY	GLY	GLY	GLY	GLY	GLY
924	ARG	ARG	ARG	ARG	ARG	ARG
925	SER	SER	SER	SER	SER	SER
926	VAL	VAL	VAL	VAL	VAL	VAL
927	ALA	ALA	ALA	ALA	ALA	ALA
928	ASP	ASP	ASP	ASP	ASP	ASP
929	LEU	LEU	LEU	LEU	LEU	LEU
930	VAL	VAL	VAL	VAL	VAL	VAL
931	CYS	CYS	CYS	CYS	CYS	CYS
932	ALA	ALA	ALA	ALA	ALA	ALA
933	GLN	GLN	GLN	GLN	GLN	GLN
934	TYR	TYR	TYR	TYR	TYR	TYR
935	TYR	TYR	TYR	TYR	TYR	TYR
936	SER	SER	SER	SER	SER	SER
937	GLY	GLY	GLY	GLY	GLY	GLY
938	VAL	VAL	VAL	VAL	VAL	VAL
939	MET	MET	MET	MET	MET	MET
940	VAL	VAL	VAL	VAL	VAL	VAL
941	LEU	LEU	LEU	LEU	LEU	LEU
942	PRO	PRO	PRO	PRO	PRO	PRO
943	GLY	GLY	GLY	GLY	GLY	GLY
944	VAL	VAL	VAL	VAL	VAL	VAL
945	VAL	VAL	VAL	VAL	VAL	VAL
946	ASP	ASP	ASP	ASP	ASP	ASP

	Amino acids					
**Position**	**DTI1**	**BP-2016**	**CBR1**	**C9822**	**VCF**	**NPPED**
947	ALA	ALA	ALA	ALA	ALA	ALA
948	GLU	GLU	GLU	GLU	GLU	GLU
949	LYS	LYS	LYS	LYS	LYS	LYS
950	LEU	LEU	LEU	LEU	LEU	LEU
951	HIS	HIS	HIS	HIS	HIS	HIS
952	MET	MET	MET	MET	MET	MET
953	TYR	TYR	TYR	TYR	TYR	TYR
954	SER	SER	SER	SER	SER	SER
955	ALA	ALA	ALA	ALA	ALA	ALA
956	SER	SER	SER	SER	SER	SER
957	LEU	LEU	LEU	LEU	LEU	LEU
958	ILE	ILE	ILE	ILE	ILE	ILE
959	GLY	GLY	GLY	GLY	GLY	GLY
960	GLY	GLY	GLY	GLY	GLY	GLY
961	MET	MET	MET	MET	MET	MET
962	VAL	VAL	VAL	ALA	**ALA**	**ALA**
963	LEU	LEU	LEU	LEU	LEU	LEU
964	GLY	GLY	GLY	GLY	GLY	GLY
965	GLY	GLY	GLY	GLY	GLY	GLY
966	PHE	PHE	PHE	PHE	PHE	PHE
967	THR	THR	THR	THR	THR	THR
968	ALA	ALA	ALA	ALA	ALA	ALA
969	ALA	ALA	ALA	ALA	ALA	ALA
970	ALA	ALA	ALA	ALA	ALA	ALA
971	ALA	ALA	ALA	ALA	ALA	ALA
972	LEU	LEU	LEU	LEU	LEU	LEU
973	PRO	PRO	PRO	PRO	PRO	PRO
974	PHE	PHE	PHE	PHE	PHE	PHE
975	SER	SER	SER	SER	SER	SER
976	TYR	TYR	TYR	TYR	TYR	TYR
977	ALA	ALA	ALA	ALA	ALA	ALA
978	VAL	VAL	VAL	VAL	VAL	VAL
979	GLN	GLN	GLN	GLN	GLN	GLN
980	ALA	ALA	ALA	ALA	ALA	ALA
981	ARG	ARG	ARG	ARG	ARG	ARG
982	LEU	LEU	LEU	LEU	LEU	LEU
983	ASN	ASN	ASN	ASN	ASN	ASN
984	TYR	TYR	TYR	TYR	TYR	TYR
985	LEU	LEU	LEU	LEU	LEU	LEU
986	ALA	ALA	ALA	ALA	ALA	ALA
987	LEU	LEU	LEU	LEU	LEU	LEU
988	GLN	GLN	GLN	GLN	GLN	GLN
989	THR	THR	THR	THR	THR	THR
990	ASP	ASP	ASP	ASP	ASP	ASP
991	VAL	VAL	VAL	VAL	VAL	VAL
992	LEU	LEU	LEU	LEU	LEU	LEU
993	GLN	GLN	GLN	GLN	GLN	GLN
994	ARG	ARG	ARG	ARG	ARG	ARG
995	ASN	ASN	ASN	ASN	ASN	ASN

	Amino acids					
**Position**	**DTI1**	**BP-2016**	**CBR1**	**C9822**	**VCF**	**NPPED**
996	GLN	GLN	GLN	GLN	GLN	GLN
997	GLN	GLN	GLN	GLN	GLN	GLN
998	LEU	LEU	LEU	LEU	LEU	LEU
999	LEU	LEU	LEU	LEU	LEU	LEU
1000	ALA	ALA	ALA	ALA	ALA	ALA
1001	GLU	GLU	GLU	GLU	GLU	GLU
1002	SER	SER	SER	SER	SER	SER
1003	PHE	PHE	PHE	PHE	PHE	PHE
1004	ASN	ASN	ASN	ASN	ASN	ASN
1005	SER	SER	SER	SER	SER	SER
1006	ALA	ALA	ALA	ALA	ALA	ALA
1007	ILE	ILE	ILE	ILE	ILE	ILE
1008	GLY	GLY	GLY	GLY	GLY	GLY
1009	ASN	ASN	ASN	ASN	ASN	ASN
1010	ILE	ILE	ILE	ILE	ILE	ILE
1011	THR	THR	THR	THR	THR	THR
1012	SER	SER	SER	SER	SER	SER
1013	ALA	ALA	ALA	ALA	ALA	ALA
1014	PHE	PHE	PHE	PHE	PHE	PHE
1015	GLU	GLU	GLU	GLU	**ASP**	GLU
1016	SER	SER	SER	SER	SER	SER
1017	VAL	VAL	VAL	VAL	VAL	VAL
1018	LYS	LYS	LYS	LYS	LYS	LYS
1019	ASP	ASP	**GLU**	**GLU**	**GLU**	**GLU**
1020	ALA	ALA	ALA	ALA	ALA	ALA
1021	ILE	ILE	ILE	ILE	ILE	ILE
1022	SER	SER	SER	SER	SER	SER
1023	GLN	GLN	GLN	GLN	GLN	GLN
1024	THR	THR	THR	THR	THR	THR
1025	SER	SER	SER	SER	SER	SER
1026	LYS	LYS	LYS	LYS	**GLN**	**GLN**
1027	GLY	GLY	GLY	GLY	GLY	GLY
1028	LEU	LEU	LEU	LEU	LEU	LEU
1029	ASN	ASN	ASN	ASN	ASN	ASN
1030	THR	THR	THR	THR	THR	THR
1031	VAL	VAL	VAL	VAL	VAL	VAL
1032	ALA	ALA	ALA	ALA	ALA	ALA
1033	HIS	HIS	HIS	HIS	HIS	HIS
1034	ALA	ALA	ALA	ALA	ALA	ALA
1035	LEU	LEU	LEU	LEU	LEU	LEU
1036	THR	THR	THR	THR	THR	THR
1037	LYS	LYS	LYS	LYS	LYS	LYS
1038	VAL	VAL	VAL	VAL	VAL	VAL
1039	GLN	GLN	GLN	GLN	GLN	GLN
1040	GLU	GLU	GLU	GLU	GLU	GLU
1041	VAL	VAL	VAL	VAL	VAL	VAL
1042	VAL	VAL	VAL	VAL	VAL	VAL
1043	ASN	ASN	ASN	ASN	ASN	ASN
1044	SER	SER	SER	SER	SER	SER

	Amino acids					
**Position**	**DTI1**	**BP-2016**	**CBR1**	**C9822**	**VCF**	**NPPED**
1045	GLN	GLN	GLN	GLN	GLN	GLN
1046	GLY	GLY	GLY	GLY	GLY	GLY
1047	ALA	ALA	ALA	ALA	ALA	ALA
1048	ALA	ALA	ALA	ALA	ALA	ALA
1049	LEU	LEU	LEU	LEU	LEU	LEU
1050	THR	THR	THR	SER	**PRO**	**SER**
1051	GLN	GLN	GLN	GLN	GLN	GLN
1052	LEU	LEU	LEU	LEU	LEU	LEU
1053	THR	THR	THR	THR	THR	THR
1054	VAL	VAL	VAL	VAL	**THR**	**ILE**
1055	GLN	GLN	GLN	GLN	GLN	GLN
1056	LEU	LEU	LEU	LEU	LEU	LEU
1057	GLN	GLN	GLN	GLN	GLN	GLN
1058	HIS	HIS	HIS	HIS	HIS	HIS
1059	ASN	ASN	ASN	ASN	ASN	ASN
1060	PHE	PHE	PHE	PHE	PHE	PHE
1061	GLN	**LYS**	GLN	GLN	GLN	GLN
1062	ALA	ALA	ALA	ALA	ALA	ALA
1063	ILE	ILE	ILE	ILE	ILE	ILE
1064	SER	SER	SER	SER	SER	SER
1065	SER	SER	SER	SER	SER	SER
1066	SER	SER	SER	SER	SER	SER
1067	ILE	ILE	ILE	ILE	ILE	**THR**
1068	ASP	ASP	ASP	ASP	ASP	ASP
1069	ASP	ASP	ASP	ASP	ASP	ASP
1070	ILE	ILE	ILE	ILE	ILE	ILE
1071	TYR	TYR	TYR	TYR	TYR	TYR
1072	SER	SER	SER	SER	**THR**	**THR**
1073	ARG	ARG	ARG	ARG	ARG	ARG
1074	LEU	LEU	LEU	LEU	LEU	LEU
1075	ASP	ASP	ASP	ASP	ASP	ASP
1076	ILE	ILE	ILE	ILE	ILE	ILE
1077	LEU	LEU	LEU	LEU	LEU	LEU
1078	SER	SER	SER	SER	SER	SER
1079	ALA	ALA	ALA	ALA	ALA	ALA
1080	ASP	ASP	ASP	ASP	ASP	ASP
1081	VAL	VAL	VAL	VAL	VAL	VAL
1082	GLN	GLN	GLN	GLN	GLN	GLN
1083	VAL	VAL	VAL	VAL	VAL	**ILE**
1084	ASP	ASP	ASP	ASP	ASP	**ASN**
1085	ARG	ARG	ARG	ARG	ARG	ARG
1086	LEU	LEU	LEU	LEU	LEU	LEU
1087	ILE	ILE	ILE	ILE	ILE	ILE
1088	ASN	**THR**	**THR**	**THR**	**THR**	**THR**
1089	GLY	GLY	GLY	GLY	GLY	VAL
1090	ARG	ARG	ARG	ARG	ARG	ARG
1091	LEU	LEU	LEU	LEU	LEU	LEU
1092	SER	SER	SER	SER	SER	SER
1093	SER	**ALA**	**ALA**	**ALA**	**ALA**	**ALA**

	Amino acids					
**Position**	**DTI1**	**BP-2016**	**CBR1**	**C9822**	**VCF**	**NPPED**
1094	LEU	LEU	LEU	LEU	LEU	LEU
1095	ASN	ASN	ASN	ASN	ASN	ASN
1096	ALA	ALA	ALA	ALA	ALA	ALA
1097	PHE	PHE	PHE	PHE	PHE	**ILE**
1098	VAL	VAL	VAL	VAL	VAL	VAL
1099	ALA	ALA	ALA	ALA	ALA	ALA
1100	GLN	GLN	GLN	GLN	GLN	GLN
1101	THR	THR	THR	THR	THR	**SER**
1102	LEU	LEU	LEU	LEU	LEU	**SER**
1103	THR	THR	THR	THR	THR	THR
1104	LYS	LYS	LYS	LYS	LYS	LYS
1105	TYR	TYR	TYR	TYR	TYR	TYR
1106	THR	THR	THR	THR	THR	**SER**
1107	GLU	GLU	GLU	GLU	GLU	GLU
1108	VAL	VAL	VAL	VAL	VAL	VAL
1109	GLN	GLN	GLN	GLN	GLN	GLN
1110	ALA	ALA	ALA	ALA	ALA	ALA
1111	SER	SER	SER	SER	SER	SER
1112	ARG	ARG	ARG	ARG	ARG	ARG
1113	LYS	LYS	LYS	LYS	**ARG**	**ARG**
1114	LEU	LEU	LEU	LEU	LEU	LEU
1115	ALA	ALA	ALA	ALA	ALA	ALA
1116	GLN	GLN	GLN	GLN	GLN	GLN
1117	GLN	GLN	GLN	GLN	GLN	GLN
1118	LYS	LYS	LYS	LYS	LYS	LYS
1119	VAL	VAL	VAL	VAL	VAL	VAL
1120	ASN	ASN	ASN	ASN	ASN	ASN
1121	GLU	GLU	GLU	GLU	GLU	GLU
1122	CYS	CYS	CYS	CYS	CYS	**SER**
1123	VAL	VAL	VAL	VAL	VAL	VAL
1124	LYS	LYS	LYS	LYS	LYS	LYS
1125	SER	SER	SER	SER	SER	SER
1126	GLN	GLN	GLN	GLN	GLN	GLN
1127	SER	SER	SER	SER	SER	**TYR**
1128	GLN	GLN	GLN	GLN	GLN	GLN
1129	ARG	ARG	ARG	ARG	ARG	ARG
1130	TYR	TYR	TYR	TYR	TYR	TYR
1131	GLY	GLY	GLY	GLY	GLY	GLY
1132	PHE	PHE	PHE	PHE	PHE	PHE
1133	CYS	CYS	CYS	CYS	CYS	CYS
1134	GLY	GLY	GLY	GLY	GLY	GLY
1135	GLY	GLY	GLY	GLY	GLY	**GLU**
1136	ASP	ASP	ASP	ASP	ASP	**HIS**
1137	GLY	GLY	GLY	GLY	GLY	GLY
1138	GLU	GLU	GLU	GLU	GLU	GLU
1139	HIS	HIS	HIS	HIS	HIS	HIS
1140	ILE	ILE	ILE	ILE	ILE	ILE
1141	PHE	PHE	PHE	PHE	PHE	PHE
1142	SER	SER	SER	SER	SER	SER

	Amino acids					
**Position**	**DTI1**	**BP-2016**	**CBR1**	**C9822**	**VCF**	**NPPED**
1143	LEU	LEU	LEU	LEU	LEU	LEU
1144	VAL	VAL	VAL	VAL	VAL	VAL
1145	GLN	GLN	GLN	GLN	GLN	GLN
1146	ALA	ALA	ALA	ALA	ALA	ALA
1147	ALA	ALA	ALA	ALA	ALA	ALA
1148	PRO	PRO	PRO	PRO	PRO	PRO
1149	GLN	GLN	GLN	GLN	GLN	GLN
1150	GLY	GLY	GLY	GLY	GLY	GLY
1151	LEU	LEU	LEU	LEU	LEU	LEU
1152	LEU	LEU	LEU	LEU	LEU	LEU
1153	PHE	PHE	PHE	PHE	PHE	PHE
1154	LEU	LEU	LEU	LEU	LEU	LEU
1155	HIS	HIS	HIS	HIS	HIS	HIS
1156	THR	THR	THR	THR	THR	THR
1157	VAL	VAL	VAL	VAL	VAL	VAL
1158	LEU	LEU	LEU	LEU	LEU	LEU
1159	VAL	VAL	VAL	VAL	VAL	**ALA**
1160	PRO	PRO	PRO	PRO	PRO	PRO
1161	GLY	GLY	GLY	GLY	GLY	GLY
1162	ASP	ASP	ASP	ASP	ASP	ASP
1163	PHE	PHE	PHE	PHE	PHE	PHE
1164	VAL	VAL	VAL	VAL	VAL	VAL
1165	ASN	ASN	ASN	ASN	ASN	ASN
1166	VAL	VAL	VAL	VAL	VAL	VAL
1167	ILE	ILE	ILE	ILE	**THR**	**THR**
1168	ALA	ALA	ALA	ALA	ALA	ALA
1169	ILE	ILE	ILE	ILE	ILE	**MET**
1170	ALA	ALA	ALA	ALA	ALA	**SER**
1171	GLY	GLY	GLY	GLY	GLY	GLY
1172	LEU	LEU	LEU	LEU	LEU	**ALA**
1173	CYS	CYS	CYS	CYS	CYS	CYS
1174	VAL	VAL	VAL	VAL	VAL	VAL
1175	ASN	ASN	ASN	ASN	ASN	ASN
1176	ASP	ASP	ASP	ASP	ASP	ASP
1177	GLU	GLU	GLU	GLU	**ASP**	**ASP**
1178	ILE	ILE	ILE	ILE	ILE	ILE
1179	ALA	ALA	ALA	ALA	ALA	**SER**
1180	LEU	LEU	LEU	LEU	LEU	**ALA**
1181	THR	THR	THR	THR	THR	**SER**
1182	LEU	LEU	LEU	LEU	LEU	**PRO**
1183	ARG	ARG	ARG	ARG	ARG	ARG
1184	GLU	GLU	GLU	GLU	GLU	GLU
1185	PRO	PRO	PRO	PRO	PRO	PRO
1186	GLY	GLY	GLY	GLY	GLY	GLY
1187	LEU	LEU	LEU	LEU	LEU	LEU
1188	VAL	VAL	VAL	VAL	VAL	VAL
1189	LEU	LEU	LEU	LEU	LEU	LEU
1190	PHE	PHE	PHE	PHE	PHE	PHE
1191	THR	THR	THR	THR	THR	THR

	Amino acids					
**Position**	**DTI1**	**BP-2016**	**CBR1**	**C9822**	**VCF**	**NPPED**
1192	HIS	HIS	HIS	HIS	HIS	HIS
1193	GLU	GLU	GLU	GLU	GLU	GLU
1194	LEU	LEU	LEU	LEU	LEU	LEU
1195	GLN	GLN	GLN	GLN	GLN	GLN
1196	ASP	ASP	ASP	ASN	**THR**	**THR**
1197	THR	THR	THR	**HIS**	**HIS**	**HIS**
1198+1				**THR**	**THR**	**LYS**
1198	ALA	ALA	ALA	ALA	ALA	**MET**
1199	THR	THR	THR	THR	THR	THR
1200	GLU	GLU	GLU	GLU	GLU	GLU
1201	TYR	TYR	TYR	TYR	TYR	TYR
1202	PHE	PHE	PHE	PHE	PHE	PHE
1203	VAL	VAL	VAL	VAL	VAL	VAL
1204	SER	SER	SER	SER	SER	SER
1205	SER	SER	SER	SER	SER	SER
1206	ARG	ARG	ARG	ARG	ARG	ARG
1207	ARG	ARG	ARG	ARG	ARG	ARG
1208	MET	MET	MET	MET	MET	MET
1209	TYR	TYR	TYR	TYR	**PHE**	**PHE**
1210	GLU	GLU	GLU	GLU	GLU	GLU
1211	PRO	PRO	PRO	PRO	PRO	PRO
1212	ARG	ARG	ARG	ARG	ARG	ARG
1213	LYS	LYS	LYS	LYS	LYS	LYS
1214	PRO	PRO	PRO	PRO	PRO	PRO
1215	THR	THR	THR	THR	THR	THR
1216	VAL	VAL	VAL	VAL	VAL	VAL
1217	GLY	GLY	GLY	GLY	**SER**	**SER**
1218	ASP	ASP	ASP	ASP	ASP	ASP
1219	PHE	PHE	PHE	PHE	PHE	PHE
1220	VAL	VAL	VAL	VAL	VAL	VAL
1221	GLN	GLN	GLN	GLN	GLN	GLN
1222	ILE	ILE	ILE	ILE	ILE	ILE
1223	GLU	GLU	GLU	GLU	GLU	GLU
1224	SER	SER	SER	SER	SER	SER
1225	CYS	CYS	CYS	CYS	CYS	CYS
1226	VAL	VAL	VAL	VAL	VAL	VAL
1227	VAL	VAL	VAL	VAL	VAL	VAL
1228	THR	THR	THR	THR	THR	**ILE**
1229	TYR	TYR	TYR	TYR	TYR	**ASN**
1230	VAL	VAL	VAL	VAL	VAL	VAL
1231	ASN	ASN	ASN	ASN	ASN	ASN
1232	LEU	LEU	LEU	LEU	LEU	LEU
1233	THR	THR	THR	THR	THR	THR
1234	ARG	ARG	ARG	ARG	ARG	ARG
1235	ASP	ASP	ASP	ASP	ASP	ASP
1236	GLN	GLN	GLN	GLN	GLN	GLN
1237	LEU	LEU	LEU	LEU	LEU	LEU
1238	PRO	PRO	PRO	PRO	PRO	PRO
1239	GLU	GLU	GLU	GLU	**ASP**	**ASP**

	Amino acids					
**Position**	**DTI1**	**BP-2016**	**CBR1**	**C9822**	**VCF**	**NPPED**
1240	VAL	VAL	VAL	VAL	VAL	VAL
1241	ILE	ILE	ILE	ILE	ILE	ILE
1242	PRO	PRO	PRO	PRO	PRO	PRO
1243	ASP	ASP	ASP	ASP	ASP	ASP
1244	TYR	TYR	TYR	TYR	TYR	TYR
1245	ILE	ILE	ILE	ILE	ILE	ILE
1246	ASP	ASP	ASP	ASP	ASP	ASP
1247	VAL	VAL	VAL	VAL	VAL	VAL
1248	ASN	ASN	ASN	ASN	ASN	ASN
1249	LYS	LYS	LYS	LYS	LYS	**HIS**
1250	THR	THR	THR	THR	THR	THR
1251	LEU	LEU	LEU	LEU	LEU	**ARG**
1252	ASP	ASP	ASP	ASP	ASP	**GLU**
1253	GLU	GLU	GLU	GLU	GLU	GLU

Table S6. The discontinuous epitope (DE) prediction of the representative sequences used in this study is shown. The color highlights in each cell correspond to the structural model available at the deposit URL. The lines between rows indicate the positions and amino acid data representing the discontinuous residues. 'NA' denotes unavailable data (information on the 3D structure of the strain in this study can be found in Files S2 and S3).

Deposit URL

DTI: https://sketchfab.com/3d-models/dti1-epitope-a4aadfe0bb9d4a81b1c48aff5c934baa

VCF: https://sketchfab.com/3d-models/vcf-epitope-56d9768ac1c44aeb99d0d0e8c10a887f

DE1

**Strain**	**DTI1**		**BP-2016**		**CBR1**		**VCF**		**NPPED**		**C9822**	
**Residues**	167		169		199		170		170		170	
**Score**	0.787		0.78		0.771		0.78		0.78		0.781	
	778	K	773	K	773	K	776	K	773	K	777	K
	779	I	774	I	774	I	777	I	774	I	778	I
	780	A	775	A	775	A	778	A	775	A	779	A
	781	P	776	P	776	P	779	P	776	P	780	P
	782	T	777	T	777	T	780	T	777	T	781	T
	783	V	778	V	778	V	781	V	778	V	782	V
	784	T	779	T	779	T	782	T	779	T	783	T
	785	G	780	G	780	G	783	G	780	G	784	G
	786	N	781	N	781	N	784	N	781	N	785	N
	787	I	782	I	782	I	785	I	782	I	786	I
	788	S	783	S	783	S	786	S	783	S	787	S
	789	I	784	I	784	I	787	I	784	I	788	I
	790	P	785	P	785	P	788	P	785	P	789	P
	791	T	786	T	786	T	789	T	786	T	790	T
	792	N	787	N	787	N	790	N	787	N	791	N
	793	F	788	F	788	F	791	F	788	F	792	F
	851	A	845	S	807	D	848	S	845	S	849	S
	854	N	846	A	808	C	849	V	846	V	850	V
	855	S	849	N	809	A	852	N	849	N	853	N
	857	L	850	S	810	T	853	S	850	S	854	S
	858	T	852	L	813	C	855	L	852	L	856	L

**Strain**	**DTI1**		**BP-2016**		**CBR1**		**VCF**		**NPPED**		**C9822**	
**Residues**	167		169		199		170		170		170	
**Score**	0.787		0.78		0.771		0.78		0.78		0.781	
	859	I	853	T	814	N	856	T	853	T	857	T
	860	S	854	I	815	G	857	I	854	I	858	I
	861	E	855	S	816	N	858	S	855	S	859	S
	862	E	856	E	817	S	859	E	856	E	860	E
	863	A	857	E	818	R	860	E	857	E	861	E
	864	L	858	A	819	C	861	A	858	A	862	A
	865	Q	859	L	820	K	862	L	859	L	863	L
	866	L	860	Q	821	Q	863	Q	860	Q	864	Q
	867	A	861	L	822	L	864	L	861	L	865	L
	868	T	862	A	823	L	865	A	862	A	866	A
	869	I	863	T	824	T	866	T	863	T	867	T
	870	S	864	I	825	Q	867	I	864	I	868	I
	871	S	865	S	826	Y	868	S	865	S	869	S
	872	F	866	S	827	T	869	S	866	S	870	S
	873	N	867	F	828	A	870	F	867	F	871	F
	874	G	868	N	829	A	871	N	868	N	872	N
	875	D	869	G	830	C	872	G	869	G	873	G
	876	G	870	D	831	K	873	D	870	D	874	D
	964	G	871	G	832	T	874	G	871	G	875	G
	965	G	944	V	833	I	947	V	944	V	948	V
	966	M	959	G	834	E	962	G	959	G	963	G
	967	V	960	G	835	S	963	G	960	G	964	G
	968	L	961	M	836	A	964	M	961	M	965	M
	969	G	962	V	838	Q	965	A	962	A	966	A
	970	G	963	L	839	L	966	L	963	L	967	L
	971	F	964	G	842	R	967	G	964	G	968	G
	972	T	965	G	843	P	968	G	965	G	969	G
	973	A	966	F	846	A	969	F	966	F	970	F
	974	A	967	T	849	N	970	T	967	T	971	T
	975	A	968	A	850	S	971	A	968	A	972	A
	976	A	969	A	852	L	972	A	969	A	973	A
	977	L	970	A	853	T	973	A	970	A	974	A
	978	P	971	A	854	I	974	A	971	A	975	A
	980	S	972	L	855	S	975	L	972	L	976	L
	981	Y	973	P	856	E	976	P	973	P	977	P
	982	A	975	S	857	E	978	S	975	S	979	S
	984	Q	976	Y	858	A	979	Y	976	Y	980	Y
	985	A	977	A	859	L	980	A	977	A	981	A
	987	L	979	Q	860	Q	982	Q	979	Q	983	Q
	988	N	980	A	861	L	983	A	980	A	984	A
	989	Y	982	L	862	A	985	L	982	L	986	L
	990	L	983	N	863	T	986	N	983	N	987	N
	991	A	984	Y	864	I	987	Y	984	Y	988	Y
	992	L	985	L	865	S	988	L	985	L	989	L
	993	Q	986	A	866	S	989	A	986	A	990	A
	994	T	987	L	867	F	990	L	987	L	991	L
	995	D	988	Q	868	N	991	Q	988	Q	992	Q
	996	V	989	T	869	G	992	T	989	T	993	T
	997	L	990	D	870	D	993	D	990	D	994	D

**Strain**	**DTI1**		**BP-2016**		**CBR1**		**VCF**		**NPPED**		**C9822**	
**Residues**	167		169		199		170		170		170	
**Score**	0.787		0.78		0.771		0.78		0.78		0.781	
	998	Q	991	V	871	G	994	V	991	V	995	V
	999	R	992	L	959	G	995	L	992	L	996	L
	1000	N	993	Q	960	G	996	Q	993	Q	997	Q
	1001	Q	994	R	961	M	997	R	994	R	998	R
	1002	Q	995	N	962	V	998	N	995	N	999	N
	1166	G	996	Q	963	L	999	Q	996	Q	1000	Q
	1167	D	997	Q	964	G	1000	Q	997	Q	1001	Q
	1168	F	1161	G	965	G	1164	G	1161	G	1165	G
	1169	V	1162	D	966	F	1165	D	1162	D	1166	D
	1170	N	1163	F	967	T	1166	F	1163	F	1167	F
	1171	V	1164	V	968	A	1167	V	1164	V	1168	V
	1172	I	1165	N	969	A	1168	N	1165	N	1169	N
	1173	A	1166	V	970	A	1169	V	1166	V	1170	V
	1174	I	1167	I	971	A	1170	T	1167	T	1171	I
	1175	A	1168	A	972	L	1171	A	1168	A	1172	A
	1176	G	1169	I	973	P	1172	I	1169	M	1173	I
	1177	L	1170	A	975	S	1173	A	1170	S	1174	A
	1178	C	1171	G	976	Y	1174	G	1171	G	1175	G
	1179	V	1172	L	977	A	1175	L	1172	A	1176	L
	1180	N	1173	C	979	Q	1176	C	1173	C	1177	C
	1181	D	1174	V	980	A	1177	V	1174	V	1178	V
	1182	E	1175	N	982	L	1178	N	1175	N	1179	N
	1183	I	1176	D	983	N	1179	D	1176	D	1180	D
	1184	A	1177	E	984	Y	1180	D	1177	D	1181	E
	1185	L	1178	I	985	L	1181	I	1178	I	1182	I
	1186	T	1179	A	986	A	1182	A	1179	S	1183	A
	1187	L	1180	L	987	L	1183	L	1180	A	1184	L
	1188	R	1181	T	988	Q	1184	T	1181	S	1185	T
	1189	E	1182	L	989	T	1185	L	1182	P	1186	L
	1190	P	1183	R	990	D	1186	R	1183	R	1187	R
	1191	G	1184	E	991	V	1187	E	1184	E	1188	E
	1192	L	1185	P	992	L	1188	P	1185	P	1189	P
	1193	V	1186	G	993	Q	1189	G	1186	G	1190	G
	1194	L	1187	L	994	R	1190	L	1187	L	1191	L
	1195	F	1188	V	995	N	1191	V	1188	V	1192	V
	1196	T	1189	L	996	Q	1192	L	1189	L	1193	L
	1197	H	1190	F	997	Q	1193	F	1190	F	1194	F
	1198	E	1191	T	1161	G	1194	T	1191	T	1195	T
	1199	L	1192	H	1162	D	1195	H	1192	H	1196	H
	1200	Q	1193	E	1163	F	1196	E	1193	E	1197	E
	1201	D	1194	L	1164	V	1197	L	1194	L	1198	L
	1202	T	1195	Q	1165	N	1198	Q	1195	Q	1199	Q
	1203	A	1196	D	1166	V	1199	T	1196	T	1200	N
	1204	T	1197	T	1167	I	1200	H	1197	H	1201	H
	1205	E	1198	A	1168	A	1201	T	1198	K	1202	T
	1206	Y	1199	T	1169	I	1202	A	1199	M	1203	A
	1207	F	1200	E	1170	A	1203	T	1200	T	1204	T
	1208	V	1201	Y	1171	G	1204	E	1201	E	1205	E
	1209	S	1202	F	1172	L	1205	Y	1202	Y	1206	Y

**Strain**	**DTI1**		**BP-2016**		**CBR1**		**VCF**		**NPPED**		**C9822**	
**Residues**	167		169		199		170		170		170	
**Score**	0.787		0.78		0.771		0.78		0.78		0.781	
	1210	S	1203	V	1173	C	1206	F	1203	F	1207	F
	1211	R	1204	S	1174	V	1207	V	1204	V	1208	V
	1213	M	1205	S	1175	N	1208	S	1205	S	1209	S
	1214	Y	1206	R	1176	D	1209	S	1206	S	1210	S
	1215	E	1208	M	1177	E	1210	R	1207	R	1211	R
	1216	P	1209	Y	1178	I	1212	M	1209	M	1213	M
	1217	R	1210	E	1179	A	1213	F	1210	F	1214	Y
	1218	K	1211	P	1180	L	1214	E	1211	E	1215	E
	1219	P	1212	R	1181	T	1215	P	1212	P	1216	P
	1220	T	1213	K	1182	L	1216	R	1213	R	1217	R
	1221	V	1214	P	1183	R	1217	K	1214	K	1218	K
	1222	G	1215	T	1184	E	1218	P	1215	P	1219	P
	1223	D	1216	V	1185	P	1219	T	1216	T	1220	T
	1224	F	1217	G	1186	G	1220	V	1217	V	1221	V
	1225	V	1218	D	1187	L	1221	S	1218	S	1222	G
	1226	Q	1219	F	1188	V	1222	D	1219	D	1223	D
	1227	I	1220	V	1189	L	1223	F	1220	F	1224	F
	1228	E	1221	Q	1190	F	1224	V	1221	V	1225	V
	1229	S	1222	I	1191	T	1225	Q	1222	Q	1226	Q
	1230	C	1223	E	1192	H	1226	I	1223	I	1227	I
	1231	V	1224	S	1193	E	1227	E	1224	E	1228	E
	1232	V	1225	C	1194	L	1228	S	1225	S	1229	S
	1233	T	1226	V	1195	Q	1229	C	1226	C	1230	C
	1234	Y	1227	V	1196	D	1230	V	1227	V	1231	V
	1235	V	1228	T	1197	T	1231	V	1228	V	1232	V
	1236	N	1229	Y	1198	A	1232	T	1229	I	1233	T
	1237	L	1230	V	1199	T	1233	Y	1230	N	1234	Y
	1238	T	1231	N	1200	E	1234	V	1231	V	1235	V
	1239	R	1232	L	1201	Y	1235	N	1232	N	1236	N
	1240	D	1233	T	1202	F	1236	L	1233	L	1237	L
	1241	Q	1234	R	1203	V	1237	T	1234	T	1238	T
	1242	L	1235	D	1204	S	1238	R	1235	R	1239	R
	1243	P	1236	Q	1205	S	1239	D	1236	D	1240	D
	1244	E	1237	L	1206	R	1240	Q	1237	Q	1241	Q
	1245	V	1238	P	1208	M	1241	L	1238	L	1242	L
	1246	I	1239	E	1209	Y	1242	P	1239	P	1243	P
	1247	P	1240	V	1210	E	1243	D	1240	D	1244	E
	1248	D	1241	I	1211	P	1244	V	1241	V	1245	V
	1249	Y	1242	P	1212	R	1245	I	1242	I	1246	I
	1250	I	1243	D	1213	K	1246	P	1243	P	1247	P
	1251	D	1244	Y	1214	P	1247	D	1244	D	1248	D
	1252	V	1245	I	1215	T	1248	Y	1245	Y	1249	Y
	1253	N	1246	D	1216	V	1249	I	1246	I	1250	I
	1254	K	1247	V	1217	G	1250	D	1247	D	1251	D
	1255	T	1248	N	1218	D	1251	V	1248	V	1252	V
	1256	L	1249	K	1219	F	1252	N	1249	N	1253	N
	1257	D	1250	T	1220	V	1253	K	1250	H	1254	K
	1258	E	1251	L	1221	Q	1254	T	1251	T	1255	T
			1252	D	1222	I	1255	L	1252	R	1256	L

**Strain**	**DTI1**		**BP-2016**		**CBR1**		**VCF**		**NPPED**		**C9822**	
**Residues**	167		169		199		170		170		170	
**Score**	0.787		0.78		0.771		0.78		0.78		0.781	
			1253	E	1223	E	1256	D	1253	E	1257	D
					1224	S	1257	E	1254	E	1258	E
					1225	C						
					1226	V						
					1227	V						
					1228	T						
					1229	Y						
					1230	V						
					1231	N						
					1232	L						
					1233	T						
					1234	R						
					1235	D						
					1236	Q						
					1237	L						
					1238	P						
					1239	E						
					1240	V						
					1241	I						
					1242	P						
					1243	D						
					1244	Y						
					1245	I						
					1246	D						
					1247	V						
					1248	N						
					1249	K						
					1250	T						
					1251	L						
					1252	D						
					1253	E						

DE2

**Epitope**	**DTI1**		**BP-2016**		**CBR1**		**VCF**		**NPPED**		**C9822**	
**Residues**	194		192		195		195		195		195	
**Score**	0.781		0.783		0.78		0.78		0.779		0.781	
	502	Q	497	Q	497	Q	500	Q	497	Q	501	Q
	503	P	498	S	498	P	501	P	498	P	502	P
	504	T	499	T	499	T	502	I	499	I	503	I
	505	S	500	S	500	S	503	S	500	S	504	S
	506	F	501	F	501	F	504	F	501	F	505	F
	507	V	502	V	502	V	505	V	502	V	506	V
	508	T	503	T	503	T	506	T	503	T	507	T
	509	L	504	L	504	L	507	L	504	L	508	L
	510	P	505	P	505	P	508	P	505	P	509	P
	511	S	506	S	506	S	509	S	506	S	510	S
	512	F	507	F	507	F	510	F	507	F	511	F
	513	N	508	N	508	N	511	N	508	N	512	N
	514	D	509	D	509	D	512	D	509	D	513	D

Epitope	DTI1		BP-2016		CBR1		VCF		NPPED		C9822	
Residues	194		192		195		195		195		195	
Score	0.781		0.783		0.78		0.78		0.779		0.781	
	515	H	510	H	510	H	513	H	510	H	514	H
	516	S	511	S	511	S	514	S	511	S	515	S
	517	F	512	F	512	F	515	F	512	F	516	F
	518	V	513	V	513	V	516	V	513	V	517	V
	519	N	514	N	514	N	517	N	514	N	518	N
	520	I	515	I	515	I	518	I	515	I	519	I
	521	T	516	T	516	T	519	T	516	T	520	T
	522	V	517	V	517	V	520	V	517	V	521	V
	523	S	518	S	518	S	521	S	518	S	522	S
	524	A	519	A	519	A	522	A	519	A	523	A
	525	A	520	A	520	A	523	S	520	S	524	S
	526	F	521	F	521	F	524	F	521	F	525	F
	527	G	522	G	522	G	525	G	522	G	526	G
	528	D	523	G	523	G	526	G	523	G	527	G
	529	H	524	H	524	H	527	H	524	H	528	Y
	530	S	525	S	525	R	528	S	525	S	529	S
	531	G	526	G	526	G	529	G	526	G	530	G
	532	A	527	A	527	A	530	A	527	A	531	A
	533	N	528	N	528	N	531	N	528	N	532	N
	534	L	529	L	529	L	532	L	529	L	533	L
	535	I	530	I	530	I	533	I	530	I	534	I
	536	A	531	A	531	A	534	A	531	A	535	A
	537	S	532	S	532	S	535	S	532	S	536	S
	538	D	533	D	533	D	536	D	533	D	537	D
	539	T	534	T	534	T	537	T	534	T	538	T
	540	T	535	T	535	T	538	T	535	T	539	T
	541	I	536	I	536	I	539	I	536	I	540	I
	542	N	537	N	537	N	540	N	537	N	541	N
	543	G	538	G	538	G	541	G	538	G	542	G
	544	F	539	F	539	F	542	F	539	F	543	F
	545	S	540	S	540	S	543	S	540	S	544	S
	546	S	541	S	541	S	544	S	541	S	545	S
	547	F	542	F	542	F	545	F	542	F	546	F
	548	C	543	C	543	R	546	C	543	C	547	C
	549	V	544	V	544	V	547	V	544	V	548	V
	550	D	545	D	545	D	548	D	545	D	549	D
	551	T	546	T	546	T	549	T	546	T	550	T
	552	R	547	R	547	R	550	R	547	R	551	R
	553	Q	548	Q	548	Q	551	Q	548	Q	552	Q
	554	F	549	F	549	F	552	F	549	F	553	F
	555	T	550	T	550	T	553	T	550	T	554	T
	556	I	551	I	551	I	554	I	551	I	555	I
	557	T	552	T	552	S	555	S	552	S	556	S
	558	L	553	L	553	R	556	L	553	L	557	L
	559	F	554	F	554	F	557	F	554	F	558	F
	560	Y	555	Y	555	Y	558	Y	555	C	559	Y
	561	N	556	N	556	N	559	N	556	N	560	N
	562	V	557	V	557	V	560	V	557	V	561	V
	563	T	558	T	558	P	561	T	558	T	562	T
	564	N	559	N	559	T	562	N	559	N	563	N

**Epitope**	**DTI1**		**BP-2016**		**CBR1**		**VCF**		**NPPED**		**C9822**	
**Residues**	194		192		195		195		195		195	
**Score**	0.781		0.783		0.78		0.78		0.779		0.781	
	565	S	560	S	560	S	563	S	560	S	564	S
	566	Y	561	Y	561	Y	564	Y	561	Y	565	Y
	567	G	562	G	562	G	565	G	562	G	566	G
	568	Y	563	Y	563	Y	566	Y	563	Y	567	Y
	569	V	564	V	564	G	567	V	564	V	568	V
	570	S	565	S	565	S	568	S	565	S	569	S
	571	K	566	K	566	K	569	K	566	K	570	K
	572	S	567	S	567	S	570	S	567	S	571	S
	573	Q	568	Q	568	Q	571	Q	568	Q	572	S
	574	D	569	D	569	G	572	D	569	D	573	D
	575	S	571	N	570	S	573	S	570	S	574	S
	576	N	572	C	571	N	574	N	571	N	575	N
	577	C	573	P	572	C	575	C	572	C	576	C
	578	P	574	F	573	P	576	P	573	P	577	P
	579	F	575	T	574	F	577	F	574	F	578	F
	580	T	576	L	575	T	578	T	575	T	579	T
	581	L	577	Q	576	L	579	L	576	L	580	L
	582	Q	578	S	577	Q	580	Q	577	Q	581	Q
	584	V	579	V	578	S	581	S	578	S	582	S
	585	N	580	N	579	V	582	V	579	V	583	V
	586	D	581	D	580	N	583	N	580	N	584	N
	587	Y	582	Y	581	D	584	D	581	D	585	D
	588	L	583	L	582	Y	585	Y	582	Y	586	Y
	589	S	584	S	583	L	586	L	583	L	587	L
	590	F	585	F	584	S	587	S	584	S	588	S
	591	S	586	S	585	F	588	F	585	F	589	F
	592	K	587	K	586	S	589	S	586	S	590	S
	593	F	588	F	587	K	590	K	587	K	591	K
	594	C	589	C	588	F	591	F	588	F	592	F
	595	V	590	V	589	C	592	C	589	C	593	C
	596	S	591	S	590	V	593	V	590	V	594	V
	597	T	592	T	591	S	594	S	591	S	595	S
	598	S	593	S	592	T	595	T	592	T	596	T
	599	L	594	L	593	S	596	S	593	S	597	S
	600	L	595	L	594	L	597	L	594	L	598	L
	601	A	596	A	595	L	598	L	595	L	599	L
	602	S	597	S	596	A	599	A	596	A	600	A
	603	A	598	A	597	S	600	S	597	S	601	S
	604	C	599	C	598	A	601	A	598	A	602	A
	605	T	600	T	599	C	602	C	599	C	603	C
	606	I	601	I	600	T	603	T	600	T	604	T
	607	D	602	D	601	I	604	I	601	I	605	I
	608	L	603	L	602	D	605	D	602	D	606	D
	609	F	604	F	603	L	606	L	603	L	607	L
	610	G	605	G	604	F	607	F	604	F	608	F
	611	H	606	Y	605	G	608	G	605	G	609	G
	612	P	607	P	606	Y	609	Y	606	Y	610	Y
	613	E	608	E	607	P	610	P	607	P	611	P
	614	F	609	F	608	E	611	D	608	E	612	E
	615	G	610	G	609	F	612	F	609	F	613	F

Epitope	DTI1		BP-2016		CBR1		VCF		NPPED		C9822	
Residues	194		192		195		195		195		195	
Score	0.781		0.783		0.78		0.78		0.779		0.781	
	616	S	611	S	610	G	613	G	610	G	614	G
	617	G	612	G	611	S	614	S	611	S	615	S
	618	V	613	V	612	G	615	G	612	G	616	G
	619	K	614	K	613	V	616	V	613	V	617	V
	620	F	615	F	614	K	617	K	614	K	618	K
	621	T	616	T	615	F	618	F	615	F	619	F
	622	S	617	S	616	T	619	T	616	T	620	T
	623	L	618	L	617	S	620	S	617	S	621	S
	624	Y	619	Y	618	L	621	L	618	L	622	L
	625	F	620	F	619	Y	622	Y	619	Y	623	Y
	626	Q	621	Q	620	F	623	F	620	F	624	F
	627	F	622	F	621	Q	624	Q	621	Q	625	Q
	628	T	623	T	622	F	625	F	622	F	626	F
	629	K	624	K	623	T	626	T	623	T	627	T
	630	G	625	G	624	K	627	K	624	K	628	K
	631	E	626	E	625	G	628	G	625	G	629	G
	632	L	627	L	626	E	629	E	626	E	630	E
	633	I	628	I	627	L	630	L	627	L	631	L
	634	T	629	T	628	I	631	I	628	I	632	I
	635	S	630	G	629	T	632	T	629	T	633	T
	636	T	631	T	630	G	633	G	630	G	634	G
	637	P	632	P	631	T	634	T	631	T	635	T
	638	K	633	K	632	P	635	P	632	P	636	P
	639	P	634	P	633	K	636	K	633	K	637	K
	640	L	635	L	634	P	637	P	634	P	638	P
	641	E	636	E	635	L	638	L	635	L	639	L
	642	G	637	G	636	E	639	E	636	E	640	E
	643	V	638	V	637	G	640	G	637	G	641	G
	644	T	639	T	638	V	641	V	638	V	642	V
	645	D	640	D	639	T	642	T	639	T	643	T
	646	V	641	V	640	D	643	D	640	D	644	D
	647	S	642	S	641	V	644	V	641	V	645	V
	648	F	643	F	642	S	645	S	642	S	646	S
	649	M	644	M	643	F	646	F	643	F	647	F
	650	T	645	T	644	M	647	M	644	M	648	M
	651	L	646	L	645	T	648	T	645	T	649	T
	652	D	647	D	646	L	649	L	646	L	650	L
	653	V	648	V	647	D	650	D	647	D	651	D
	655	T	650	T	648	V	651	V	648	V	652	V
	657	Y	652	Y	650	T	653	T	650	T	654	T
	658	T	653	T	652	Y	655	Y	652	Y	656	Y
	659	I	654	I	653	T	656	T	653	T	657	T
	660	Y	655	Y	654	I	657	I	654	I	658	I
	661	G	662	I	655	Y	658	Y	655	Y	659	Y
	662	F	663	I	656	G	659	G	656	G	660	G
	667	I	664	T	657	F	660	F	657	F	661	F
	668	I	665	L	662	I	665	V	662	I	666	I
	669	T	666	T	663	I	666	I	663	I	667	I
	670	L	667	N	664	T	667	T	664	T	668	T

**Epitope**	**DTI1**		**BP-2016**		**CBR1**		**VCF**		**NPPED**		**C9822**	
**Residues**	194		192		195		195		195		195	
**Score**	0.781		0.783		0.78		0.78		0.779		0.781	
	671	T	668	S	665	L	668	L	665	L	669	L
	672	N	669	S	666	T	669	T	666	T	670	T
	673	S	670	F	667	N	670	N	667	N	671	N
	674	S	671	L	668	S	671	S	668	S	672	S
	675	F	672	A	669	S	672	S	669	S	673	S
	676	L	673	G	670	F	673	F	670	F	674	F
	677	A	674	V	671	L	674	L	671	L	675	L
	678	G	675	Y	672	A	675	A	672	A	676	A
	679	V	676	Y	673	G	676	G	673	G	677	G
	680	Y	677	T	674	V	677	V	674	V	678	V
	681	Y	678	S	675	Y	678	Y	675	Y	679	Y
	682	T	679	D	676	Y	679	Y	676	Y	680	Y
	683	S	680	S	677	T	680	T	677	T	681	T
	684	D	681	G	678	S	681	S	678	S	682	S
	685	S	682	Q	679	D	682	D	679	D	683	D
	686	G	683	L	680	S	683	S	680	S	684	S
	687	Q	684	L	681	G	684	G	681	G	685	G
	688	L	685	A	682	Q	685	Q	682	Q	686	Q
	689	L	686	F	683	L	686	L	683	L	687	L
	690	A	687	K	684	L	687	L	684	L	688	L
	691	F	688	N	685	A	688	A	685	A	689	A
	692	K	689	V	686	F	689	F	686	F	690	F
	693	N	690	T	687	K	690	K	687	K	691	K
	694	V	691	S	688	N	691	N	688	N	692	N
	695	T	692	G	689	V	692	V	689	V	693	V
	696	S	693	A	690	T	693	T	690	T	694	T
	697	G	694	I	691	S	694	S	691	S	695	S
	698	A	695	Y	692	G	695	G	692	G	696	G
	699	I	696	S	693	A	696	A	693	A	697	A
	700	Y	697	V	694	V	697	V	694	V	698	V
	701	S			695	Y	698	Y	695	Y	699	Y
	702	V			696	S	699	S	696	S	700	S
					697	V	700	V	697	V	701	V

DE3

**Epitope**	**DTI1**		**BP-2016**		**CBR1**		**VCF**		**NPPED**		**C9822**	
**Residues**	31		31		NA		49		31		54	
**Score**	0.708		0.705		NA		0.679		0.705		0.671	
	812	D	807	D			810	D	807	D	811	D
	813	C	808	C			811	C	808	C	812	C
	814	A	809	A			812	A	809	A	813	A
	815	T	810	T			813	T	810	T	814	T
	818	C	813	C			815	V	813	C	816	V
	819	N	814	N			816	C	814	N	817	C
	820	G	815	G			817	N	815	G	818	N
	821	N	816	N			818	G	816	N	819	G
	822	S	817	S			819	N	817	S	820	N
	823	R	818	R			820	S	818	R	821	S
	824	C	819	C			821	R	819	C	822	R

**Epitope**	**DTI1**		**BP-2016**		**CBR1**	**VCF**		**NPPED**		**C9822**	
**Residues**	31		31		NA	49		31		54	
**Score**	0.708		0.705		NA	0.679		0.705		0.671	
	825	K	820	K		822	C	820	K	823	C
	826	Q	821	Q		823	K	821	Q	824	K
	827	L	822	L		824	Q	822	L	825	Q
	828	L	823	L		825	L	823	L	826	L
	829	T	824	T		826	L	824	T	827	L
	830	Q	825	Q		827	T	825	Q	828	T
	831	Y	826	Y		828	Q	826	Y	829	Q
	832	T	827	T		829	Y	827	T	830	Y
	833	A	828	A		830	T	828	A	831	T
	834	A	829	A		831	A	829	A	832	A
	835	C	830	C		832	A	830	C	833	A
	836	K	831	K		833	C	831	K	834	C
	837	T	832	T		834	K	832	T	835	K
	838	I	833	I		835	T	833	I	836	T
	839	E	834	E		836	I	834	E	837	I
	840	S	835	S		837	E	835	S	838	E
	841	A	836	A		838	S	836	A	839	S
	843	Q	838	Q		839	A	838	Q	840	A
	844	L	839	L		841	Q	839	L	842	Q
	847	R	842	R		842	L	842	R	843	L
						845	R			846	R
						1064	Q			1065	Q
						1065	A			1066	A
						1073	I			1070	S
						1077	L			1071	I
						1078	D			1072	D
						1079	I			1074	I
						1080	L			1075	Y
						1081	S			1076	S
						1082	A			1078	L
						1083	D			1079	D
						1084	V			1080	I
						1085	Q			1081	L
						1086	V			1082	S
						1087	D			1083	A
						1088	R			1084	D
						1090	I			1085	V
						1091	T			1086	Q
										1087	V
										1088	D
										1089	R
										1091	I
										1092	T

DE4

Epitope	DTI1		BP-2016		CBR1		VCF		NPPED		C9822	
Residues	76		76		77		77		77		77	
Score	0.675		0.675		0.676		0.671		0.676		0.67	
	287	I	287	I	287	M	290	I	287	I	291	I
	288	P	288	P	288	P	291	P	288	P	292	P
	289	K	289	K	289	K	292	K	289	K	293	K
	290	I	290	I	290	I	293	I	290	I	294	I
	292	G	292	G	292	G	295	G	292	G	296	G
	293	L	293	L	293	L	296	L	293	L	297	L
	294	G	294	G	294	G	297	G	294	G	298	G
	295	Q	295	Q	295	Q	298	Q	295	Q	299	Q
	329	N	324	N	324	D	327	N	324	D	328	D
	330	D	325	D	325	D	328	D	325	D	329	D
	332	S	326	T	326	T	329	T	326	T	330	T
	333	V	327	S	327	A	330	S	327	S	331	S
	334	I	328	V	328	V	331	V	328	V	332	V
	335	L	329	I	329	I	332	I	329	I	333	I
	336	A	330	L	330	L	333	L	330	L	334	L
	337	E	331	A	331	A	334	A	331	A	335	A
	338	G	332	E	332	E	335	E	332	E	336	E
	339	S	333	G	333	G	336	G	333	G	337	G
	347	G	334	S	334	S	337	S	334	S	338	S
	348	T	342	G	342	G	345	G	342	G	346	G
	349	N	343	T	343	T	346	T	343	T	347	T
	351	S	344	N	344	N	347	N	344	N	348	N
	353	V	346	S	346	S	349	S	346	S	350	S
	354	C	348	V	348	V	351	V	348	V	352	V
	355	S	349	C	349	C	352	C	349	C	353	C
	356	N	350	S	350	S	353	S	350	S	354	S
	357	S	351	N	351	N	354	N	351	N	355	N
	358	S	352	S	352	S	355	S	352	S	356	S
	359	D	353	S	353	S	356	S	353	S	357	S
	360	P	354	D	354	D	357	N	354	D	358	D
	361	H	355	P	355	P	358	P	355	P	359	P
	362	S	356	H	356	H	359	H	356	H	360	H
	363	A	357	S	357	L	360	L	357	L	361	S
	364	I	358	A	358	T	361	A	358	A	362	A
	365	F	359	I	359	T	362	T	359	T	363	T
	366	A	360	F	360	F	363	F	360	F	364	F
	367	I	361	A	361	A	364	P	361	A	365	A
	368	P	362	I	362	I	365	I	362	I	366	I
	369	L	363	P	363	P	366	P	363	P	367	P
	370	G	364	L	364	L	367	L	364	L	368	L
	371	A	365	G	365	G	368	G	365	G	369	G
	372	T	366	A	366	A	369	A	366	A	370	A
	373	Q	367	T	367	T	370	T	367	T	371	T
	374	V	368	Q	368	Q	371	Q	368	Q	372	Q
	375	P	369	V	369	V	372	V	369	V	373	V
	376	Y	370	P	370	P	373	P	370	P	374	P
	377	Y	371	Y	371	Y	374	Y	371	Y	375	Y
	378	C	372	Y	372	Y	375	Y	372	Y	376	Y

**Epitope**	**DTI1**		**BP-2016**		**CBR1**		**VCF**		**NPPED**		**C9822**	
**Residues**	76		76		77		77		77		77	
**Score**	0.675		0.675		0.676		0.671		0.676		0.67	
	379	F	373	C	373	C	376	C	373	C	377	C
	381	K	374	F	374	F	377	F	374	F	378	F
	382	V	376	K	376	K	379	K	376	K	380	K
	383	D	377	V	377	V	380	G	377	V	381	V
	384	T	378	D	378	D	381	D	378	D	382	D
	385	Y	379	T	379	T	382	T	379	T	383	T
	386	N	380	Y	380	Y	383	Y	380	Y	384	Y
	387	S	381	N	381	N	384	N	381	N	385	N
	388	T	382	S	382	S	385	S	382	S	386	S
	389	V	383	T	383	T	386	T	383	T	387	S
	390	Y	384	V	384	V	387	V	384	V	388	V
	391	K	385	Y	385	Y	388	Y	385	Y	389	Y
	392	F	386	K	386	K	389	K	386	K	390	K
	395	V	387	F	387	F	390	F	387	L	391	F
	396	L	390	V	390	V	393	V	390	V	394	V
	430	N	391	L	391	L	394	L	391	L	395	L
	431	F	425	N	425	N	428	N	425	N	429	N
	432	T	426	F	426	F	429	F	426	F	430	F
	433	G	427	T	427	T	430	T	427	T	431	T
	434	H	428	G	428	G	431	G	428	G	432	G
	435	G	429	H	429	H	432	H	429	H	433	H
	436	I	431	T	430	G	433	G	430	G	434	G
	437	D	432	D	431	T	434	T	431	T	435	T
	438	G	433	G	432	D	435	D	432	D	436	D
	439	D	434	D	433	G	436	D	433	D	437	D
	440	V	435	V	434	D	437	D	434	D	438	D
	441	S	436	S	435	V	438	V	435	V	439	V
	442	G	437	G	436	S	439	S	436	S	440	S
					437	G	440	G	437	G	441	G

DE5

**Epitope**	**DTI1**		**BP-2016**		**CBR1**		**VCF**		**NPPED**		**C9822**	
**Residues**	114		115		115		117		115		118	
**Score**	0.666		0.667		0.667		0.667		0.667		0.666	
	31	N	31	N	31	N	31	N	31	N	31	N
	32	F	32	F	32	F	32	F	32	F	32	F
	33	R	33	R	33	R	33	R	33	R	33	R
	34	R	34	R	34	R	34	R	34	R	34	R
	35	F	35	F	35	F	35	F	35	F	35	F
	36	F	36	F	36	F	36	F	36	F	36	F
	37	S	37	S	37	S	37	S	37	S	37	S
	38	K	38	K	38	K	38	K	38	K	38	K
	39	F	39	F	39	F	39	F	39	F	39	F
	41	V	41	V	41	V	41	V	41	V	41	V
	42	Q	42	Q	42	Q	42	Q	42	Q	42	Q
	43	S	43	A	43	A	43	A	43	A	43	A
	44	P	44	P	44	P	44	P	44	P	44	P
	45	A	45	A	45	A	45	A	45	A	45	A

**Epitope**	**DTI1**		**BP-2016**		**CBR1**		**VCF**		**NPPED**		**C9822**	
**Residues**	114		115		115		117		115		118	
**Score**	0.666		0.667		0.667		0.667		0.667		0.666	
	46	V	46	V	46	V	46	V	46	V	46	V
	52	Y	52	Y	52	Y	52	Y	52	Y	52	Y
	53	L	53	L	53	L	53	L	53	L	53	L
	54	P	54	P	54	P	54	P	54	P	54	P
	55	T	55	T	55	I	55	I	55	I	55	I
	56	G	56	G	56	G	56	G	56	G	56	G
	57	E	57	E	57	E	57	E	57	E	57	E
	58	N	58	N	58	N	58	N	58	N	58	N
	59	Q	59	Q	59	Q	59	Q	59	Q	59	Q
	60	G	60	G	60	G	60	G	60	G	60	G
	61	V	61	V	61	V	61	V	61	V	61	V
	62	N	62	N	62	N	62	N	62	N	62	N
	63	S	63	S	63	S	63	S	63	S	63	S
	64	T	64	T	64	T	64	T	64	T	64	T
	65	W	65	W	65	W	65	W	65	W	65	W
	66	Y	66	Y	66	Y	66	Y	66	Y	66	Y
	67	C	67	C	67	C	67	C	67	C	67	C
	68	A	68	A	68	A	68	A	68	A	68	A
	69	G	69	G	69	G	69	G	69	G	69	G
	70	Q	70	Q	70	Q	70	Q	70	Q	70	Q
	71	H	71	H	71	H	71	H	71	H	71	H
	72	P	72	S	72	P	72	P	72	P	72	P
	73	T	73	T	73	T	73	T	73	T	73	T
	74	A	74	A	74	A	74	A	74	A	74	A
	75	S	75	S	75	S	75	S	75	S	75	S
	76	G	76	G	76	G	76	G	76	G	76	G
	77	V	77	V	77	V	77	V	77	V	77	V
	78	H	78	H	78	H	78	H	78	H	78	H
	81	F	80	I	80	I	81	F	80	I	81	F
	82	L	81	F	81	F	82	L	81	F	82	L
	83	S	82	L	82	L	83	S	82	L	83	S
	84	H	83	S	83	S	84	H	83	S	84	H
	85	I	84	H	84	H	85	I	84	H	85	I
	86	R	85	I	85	I	86	R	85	I	86	R
	87	G	86	R	86	R	87	G	86	R	87	G
	88	G	87	G	87	G	88	G	87	G	88	G
	89	H	88	G	88	G	89	H	88	G	89	H
	90	G	89	H	89	H	90	G	89	H	90	G
	91	F	90	G	90	G	91	F	90	G	91	F
	97	Q	91	F	91	F	97	Q	91	F	97	Q
	98	E	97	Q	97	Q	98	E	97	Q	98	E
	99	P	98	E	98	E	99	P	98	E	99	P
	112	A	99	P	99	P	112	A	99	P	112	A
	113	T	112	A	112	A	113	T	112	A	113	T
	114	N	113	T	113	T	114	N	113	T	114	N
	115	G	114	N	114	N	115	G	114	N	115	G
	116	N	115	G	115	G	116	N	115	G	116	N
	117	T	116	N	116	N	117	T	116	N	117	T
	118	N	117	T	117	T	118	N	117	T	118	N

**Epitope**	**DTI1**		**BP-2016**		**CBR1**		**VCF**		**NPPED**		**C9822**	
**Residues**	114		115		115		117		115		118	
**Score**	0.666		0.667		0.667		0.667		0.667		0.666	
	156	A	118	N	118	N	156	A	118	N	156	A
	157	H	156	A	156	A	157	Y	156	A	157	H
	158	M	157	H	157	H	158	M	157	H	158	M
	159	S	158	M	158	M	159	S	158	M	159	S
	160	E	159	S	159	S	160	E	159	S	160	E
	161	H	160	E	160	E	161	H	160	E	161	H
	162	S	161	H	161	H	162	S	161	H	162	S
	163	V	162	S	162	S	163	V	162	S	163	V
	190	S	163	V	163	V	190	S	163	V	190	S
	194	T	190	S	190	S	194	T	190	S	194	T
	196	C	194	T	194	T	196	C	194	T	196	C
	197	Y	196	C	196	C	197	Y	196	C	197	Y
	198	N	197	Y	197	Y	198	N	197	Y	198	N
	199	S	198	N	198	N	199	S	198	N	199	S
	200	G	199	S	199	S	200	G	199	S	200	G
	201	G	200	G	200	G	201	G	200	G	201	G
	202	C	201	G	201	G	202	C	201	G	202	C
	203	A	202	C	202	C	203	A	202	C	203	A
	204	M	203	A	203	A	204	M	203	A	204	M
	205	Q	204	M	204	M	205	Q	204	M	205	Q
	206	Y	205	Q	205	Q	206	Y	205	Q	206	Y
	207	V	206	Y	206	Y	207	V	206	Y	207	V
	208	Y	207	V	207	V	208	Y	207	V	208	Y
	209	E	208	Y	208	Y	209	E	208	Y	209	E
	210	P	209	E	209	E	210	P	209	E	210	P
	211	T	210	P	210	P	211	T	210	P	211	T
	212	Y	211	T	211	T	212	Y	211	T	212	Y
	213	Y	212	Y	212	Y	213	Y	212	Y	213	Y
	214	M	213	Y	213	Y	214	M	213	Y	214	M
	215	L	214	M	214	M	215	L	214	M	215	L
	216	N	215	L	215	L	216	N	215	L	216	N
	217	V	216	N	216	N	217	V	216	N	217	V
	218	T	217	V	217	V	218	T	217	V	218	T
	219	S	218	T	218	T	219	S	218	T	219	S
	220	A	219	S	219	S	220	A	219	S	220	A
	221	G	220	A	220	A	221	G	220	A	221	G
	222	E	221	G	221	G	222	E	221	G	222	E
	223	D	222	E	222	K	223	A	222	E	223	D
	224	G	223	D	223	D	224	G	223	D	224	G
	225	I	224	G	224	G	225	I	224	G	225	I
	226	S	225	I	225	I	226	S	225	I	226	S
	227	Y	226	S	226	S	227	Y	226	S	227	Y
	228	Q	227	Y	227	Y	228	Q	227	Y	228	Q
	229	P	228	Q	228	Q	229	P	228	Q	229	P
	230	C	229	P	229	P	230	C	229	P	230	C
	231	T	230	C	230	C	231	T	230	C	231	T
	232	A	231	T	231	T	232	A	231	T	232	A
	233	N	232	A	232	A	233	T	232	A	233	N
	234	C	233	N	233	N	234	R	233	N	234	T

**Epitope**	**DTI1**		**BP-2016**		**CBR1**		**VCF**		**NPPED**		**C9822**	
**Residues**	114		115		115		117		115		118	
**Score**	0.666		0.667		0.667		0.667		0.667		0.666	
	313	N	234	C	234	C	235	E	234	C	235	T
	314	G	308	N	308	N	236	Y	308	N	236	G
			309	G	309	G	237	C	309	G	237	R
							311	N			238	C
							312	G			312	N
											313	G

DE6

**Epitope**	**DTI1**		**BP-2016**		**CBR1**		**VCF**		**NPPED**		**C9822**
**Residues**	20		23		20		6		23		NA
**Score**	0.625		0.627		0.62		0.592		0.628		NA
	1066	Q	1061	K	1061	Q	1069	S	1061	Q	
	1067	A	1062	A	1062	A	1070	I	1062	A	
	1071	S	1066	S	1066	S	1071	D	1066	S	
	1072	I	1067	I	1067	I	1074	Y	1067	T	
	1073	D	1068	D	1068	D	1075	T	1068	D	
	1075	I	1070	I	1070	I	1076	R	1070	I	
	1079	L	1071	Y	1074	L			1071	Y	
	1080	D	1072	S	1075	D			1072	T	
	1081	I	1073	R	1076	I			1073	R	
	1082	L	1074	L	1077	L			1074	L	
	1083	S	1075	D	1078	S			1075	D	
	1084	A	1076	I	1079	A			1076	I	
	1085	D	1077	L	1080	D			1077	L	
	1086	V	1078	S	1081	V			1078	S	
	1087	Q	1079	A	1082	Q			1079	A	
	1088	V	1080	D	1083	V			1080	D	
	1089	D	1081	V	1084	D			1081	V	
	1090	R	1082	Q	1085	R			1082	Q	
	1092	I	1083	V	1087	I			1083	I	
	1093	N	1084	D	1088	T			1084	N	
			1085	R					1085	R	
			1087	I					1087	I	
			1088	T					1088	T	

DE7

**Epitope**	**DTI1**		**BP-2016**		**CBR1**		**VCF**		**NPPED**		**C9822**	
**Residues**	12		12		12		12		12		12	
**Score**	0.623		0.619		0.62		0.621		0.622		0.62	
	884	G	879	G	879	G	882	G	879	G	883	G
	886	S	881	S	881	S	884	S	881	S	885	S
	887	V	882	V	882	V	885	V	882	V	886	V
	889	D	884	D	884	D	887	E	884	E	888	D
	890	P	885	P	885	P	888	P	885	P	889	P
	891	A	886	A	886	A	889	A	886	A	890	A
	892	S	887	S	887	S	890	S	887	S	891	S
	893	D	888	G	888	G	891	G	888	G	892	G

	894	R	889	R	889	R	892	R	889	R	893	R
	895	V	890	V	890	V	893	V	890	V	894	V
	896	V	891	V	891	V	894	V	891	V	895	V
	897	Q	892	Q	892	Q	895	H	892	H	896	H

DE8

Epitope	DTI1		BP-2016	CBR1	VCF	NPPED
**Residues**	12		NA	NA	NA	NA
**Score**	0.623		NA	NA	NA	NA
	301	Q				
	302	T				
	305	G				
	306	E				
	307	D				
	308	L				
	309	K				
	310	S				

References

1. Temeeyasen, G.; Srijangwad, A.; Tripipat, T.; Tipsombatboon, P.; Piriyapongsa, J.; Phoolcharoen, W.; Chuanasa, T.; Tantituvanont, A.; Nilubol, D. Genetic diversity of ORF3 and spike genes of porcine epidemic diarrhea virus in Thailand. Infect. Genet. Evol. 2014, 21, 205–213. https://doi.org/10.1016/j.meegid.2013.11.001.
2. Pan, Y.; Tian, X.; Li, W.; Zhou, Q.; Wang, D.; Bi, Y.; Chen, F.; Song, Y. Isolation and characterization of a variant porcine epidemic diarrhea virus in China. Virol. J. 2012, 9, 195. https://doi.org/10.1186/1743-422X-9-195.
3. Zhao, P.D.; Tan, C.; Dong, Y.; Li, Y.; Shi, X.; Bai, J.; Jiang, P. Genetic variation analyses of porcine epidemic diarrhea virus isolated in mid-eastern China from 2011 to 2013. *Can. J. Vet. Res.* 2015, *79*, 8–15.
