# Supplementary material for: Detection and Phylogenetic Analysis of an Exotic Strain of Porcine Epidemic Diarrhea Virus and Its Effect on an Affected Herd Immunized Against the Endemic Strain in Thailand
Source: Animals (Basel). 2025 Jan 15;15(2):225. doi: 10.3390/ani15020225 (PMC11759134; doi:10.3390/ani15020225)
Supplement: Supplementary file 1 [file animals-15-00225-s001.zip › 3.Supplementary Material File S2. (DTI1_Figure_Web_Browser).html]

Simple Chimera Viewer
